# DNA-Mimicking Metal–Organic Frameworks with Accessible Adenine Faces for Complementary Base Pairing

**DOI:** 10.1021/jacsau.1c00516

**Published:** 2022-02-07

**Authors:** Santanu Chand, Othman Alahmed, Walaa S. Baslyman, Avishek Dey, Somayah Qutub, Ranajit Saha, Yuh Hijikata, Manal Alaamery, Niveen M. Khashab

**Affiliations:** ‡ Smart Hybrid Materials (SHMs) Laboratory, Advanced Membranes and Porous Materials Center, Physical Science and Engineering Division, King Abdullah University of Science and Technology (KAUST), Thuwal 23955-6900, Kingdom of Saudi Arabia; § Institute for Chemical Reaction Design and Discovery (WPI-ICReDD), Hokkaido University, Sapporo, Hokkaido 001-0021, Japan; ⊥ Developmental Medicine Department, King Abdullah Interna-tional Medical Research Center, King Saud Bin Abdulaziz University for Health Sciences, Ministry of National Guard-Health Affairs (MNG-HA), Riyadh 11481, Kingdom of Saudi Arabia

**Keywords:** DNA delivery, BioMOF, metal organic framework, nucleic acid loading, ssDNA

## Abstract

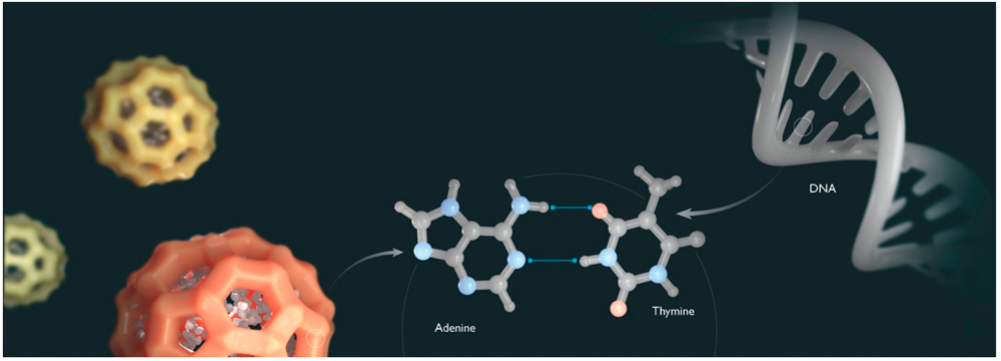

Biologically derived metal–organic frameworks (Bio-MOFs) are significant, as they can be used in cutting-edge biomedical applications such as targeted gene delivery. Herein, adenine (Ade) and unnatural amino acids coordinate with Zn^2+^ to produce biocompatible frameworks, KBM-1 and KBM-2, with extremely defined porous channels. They feature an accessible Watson–Crick Ade face that is available for further hydrogen bonding and can load single-stranded DNA (ssDNA) with 13 and 41% efficiency for KBM-1 and KBM-2, respectively. Treatment of these frameworks with thymine (Thy), as a competitive guest for base pairing with the Ade open sites, led to more than 50% reduction of ssDNA loading. Moreover, KBM-2 loaded Thy-rich ssDNA more efficiently than Thy-free ssDNA. These findings support the role of the Thy-Ade base pairing in promoting ssDNA loading. Furthermore, theoretical calculations using the self-consistent charge density functional tight-binding (SCC-DFTB) method verified the role of hydrogen bonding and van der Waals type interactions in this host–guest interface. KBM-1 and KBM-2 can protect ssDNA from enzymatic degradation and release it at acidic pH. Most importantly, these biocompatible frameworks can efficiently deliver genetic cargo with retained activity to the cell nucleus. We envisage that this class of Bio-MOFs can find immediate applicability as biomimics for sensing, stabilizing, and delivering genetic materials.

## Introduction

Prospective utilization of metal–organic frameworks (MOFs) for biomedical and pharmaceutical applications has progressed significantly over the past decade. The major impediments toward translating this revolutionary class of smart assemblies include materials’ toxicology, colloidal stability, loading efficacy, and commercial scalability.^1−4^ Biologically compatible MOFs have been designed and synthesized to circumvent these major obstacles starting with the nontoxic iron III carboxylate MOFs (MIL family) that were efficiently used for drug delivery and imaging.^5−7^ Zirconium-based MOFs with optimal stability toward hydrolysis and low toxicity were also successfully employed for drug delivery and enzyme encapsulation.^8−10^ The biomimetic in situ approach utilizing zeolitic imidazolate frameworks (ZIFs) to encapsulate bioactive molecules, proteins, and genetic materials has lately witnessed a tremendous spur due to the straight foreword assembly of ZIFs in aqueous media without the need for heating.^11−17^ However, the possible toxicity of the building blocks and limited colloidal stability, especially when considered in a pharmacokinetic setting, have halted further efforts for the broader clinical translation of these systems.

Generally, the desired features of nanocarriers for biomolecule delivery are efficient encapsulation/intercalation, high cell internalization, and spatiotemporal-controlled release into the targeted intracellular compartment. Biocompatible smart hybrid materials with competent cell permeability, high stability and appropriate pharmacokinetics have been reported for delivering drug/cargo into specific cells.^18,19^ Prospective synthetic systems should be nature-derived carriers as they gratify most of these key features, such as water solubility, biocompatibility, and high cellular uptake efficiency with marginal toxicity.

Recently, biomolecules such as peptides, sugars, curcumin, and nucleobases have emerged as promising building blocks for constructing more biologically derived MOFs (Bio-MOFs) or metal-biomolecule frameworks (MBioFs).^20−26^ Mimicking natural designs using adenine (Ade) as a building block is a great approach to achieve natural nucleobase hydrogen bonding reminiscence of the Watson–Crick double-stranded DNA assembly. However, most of the reported Ade-based MOFs had the Watson–Crick face of Ade completely occupied by coordination bonds and consequently not available for nucleobase pairing assembly.^27,28^ More recently, Stylianou and co-workers elegantly reported nucleobase pairing via a novel Bio-MOF with an unobstructed Ade face, in which the guest Thy can be dimerized upon light irradiation.^29^ This inventive concept paved the way for a new generation of DNA-inspired biocompatible frameworks.

**Figure 1:**
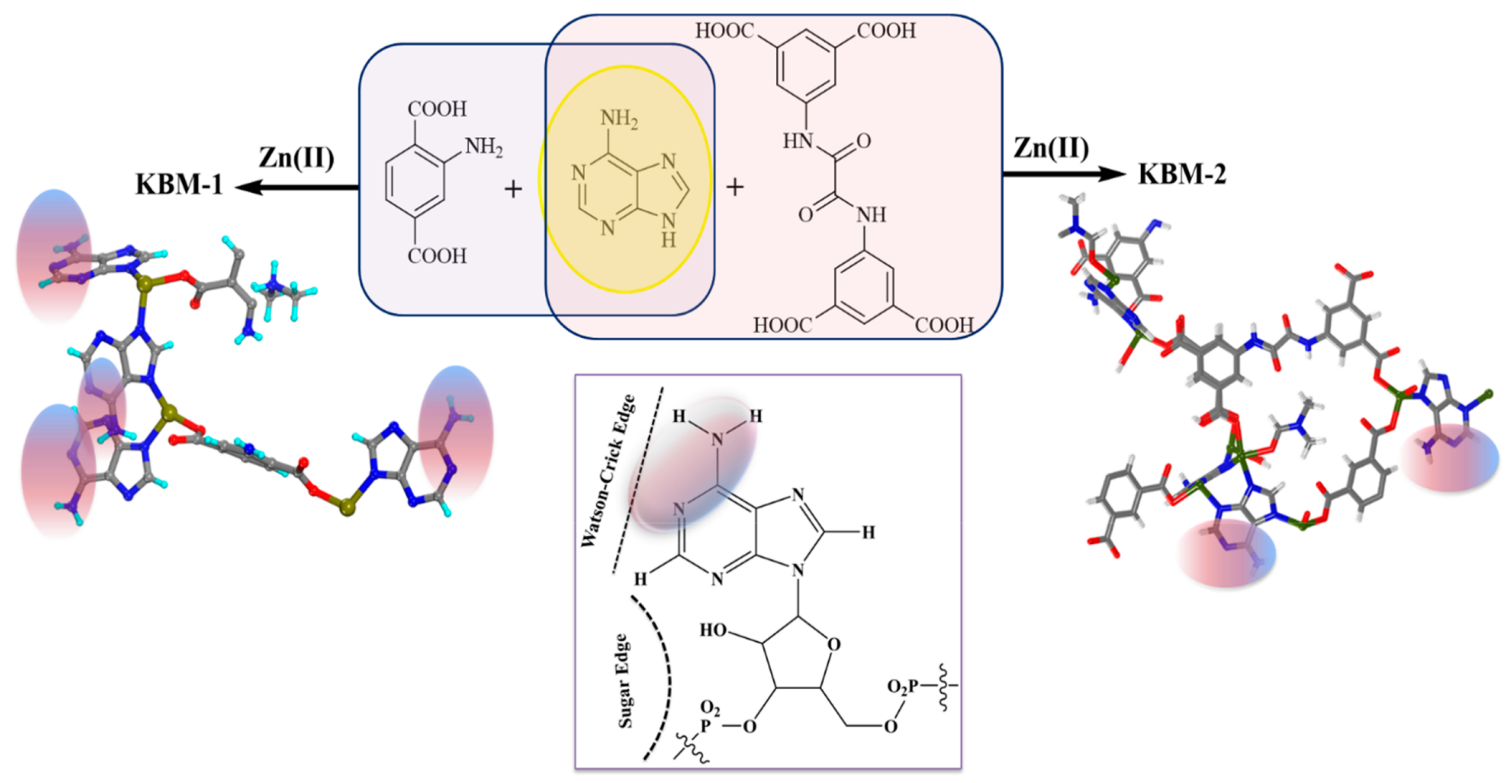
Synthetic route for KBM-1 and KBM-2. (Left) Asymmetric unit of KBM-1. (Right) Asymmetric unit of KBM-2. (Color code: carbon, gray; hydrogen, cyan/white; oxygen, red; nitrogen, blue; zinc, dark yellow.) Representation of Watson–Crick edge (shadowed) and sugar edge present in a DNA molecule.

The synergy of MOF-DNA conjugates has shown very promising results for biomedical translation.^30−37^ For example, single-stranded oligonucleotide (ssDNA) therapy has achieved remarkable success with more recent efforts addressing immune-resistant bacteria and CRISPR-Cas technology.^38^ Moreover, another ssDNA, namely, the antiproliferating cell nuclear antigen (PCNA), which is a DNA replication inhibitor, has shown excellent results in limiting cell proliferation.^39,40^ Most synthetic gene delivery vectors developed so far have been prepared on the basis of the more stable and relatively easy to handle double-stranded DNA (dsDNA).^41^ ssDNA is known to have significant flexibility, shorter length, and higher tissue penetrability than dsDNA, making it hard to condense in targeted delivery vehicles.^42^ Successful reports on packaging ssDNA depended mainly on peptide-conjugated vectors,^43,44^ cationic polymers,^45^ and inorganic nanoparticles.^46−48^ Nonetheless, the limited biocompatibility and longtime stability have halted the broader biomedical translation of these systems. Herein, we present two DNA-mimicking BioMOFs that can be easily prepared from the coordination of adenine and amino or amido dicarboxylic acid to give KAUST bioMOF-1 (KBM-1) and KAUST bioMOF-2 (KBM-2), respectively (Figure 1). KBM-1 and KBM-2 have excellent biocompatibility and aqueous stability. Most importantly, these frameworks have an open Ade Watson–Crick face that can readily load single-stranded DNA (ssDNA) aided by complementary nucleobase pairing. KBM-1 and KBM-2 can load ssDNA in 13 and 41%, respectively. The nature of the interactions promoting the loading was studied by competitive binding as when free thymine (Thy) was added to both frameworks before ssDNA loading, a reduction of more than 50% was calculated in the loading capacity. Moreover, KBM-2 can load Thy-rich ssDNA (poly CT) more efficiently than Thy-free ssDNA (poly CA). Theoretical studies on both frameworks further confirmed the role of hydrogen bonding and van der Waals type interactions in the loading of the ssDNA guest molecules. KBM-1 and KBM-2 can effectively stabilize ssDNA and protect it from enzymatic degradation. Most importantly, in vitro studies proved the ability of KBM-2 to successfully deliver ssDNA to the cell nucleus with retained activity. To the best of our knowledge, this is the first report of ssDNA loading and condensation on BioMOFs assisted by complementary base pairing.

## Results and Discussion

KBM-1 is anionic in nature with the molecular formula {[Me_2_NH_2_][Zn_3_(Ade)_4_(NH_2_BDC)_1.5_]·*x*Guest}_*n*_ having an charge balancing counter cations in its pores. It crystallizes in the symmetric orthorhombic crystal system with space group *Pbcn* (Table S1). On the other hand, KBM-2 is a neutral framework with the molecular formula [Zn_8_(Ade)_4_(L)_3_]·*x*Guest}_*n*_, and it crystallizes in the triclinic crystal system with space group *P*1̅ (Table S2).

**Figure 2:**
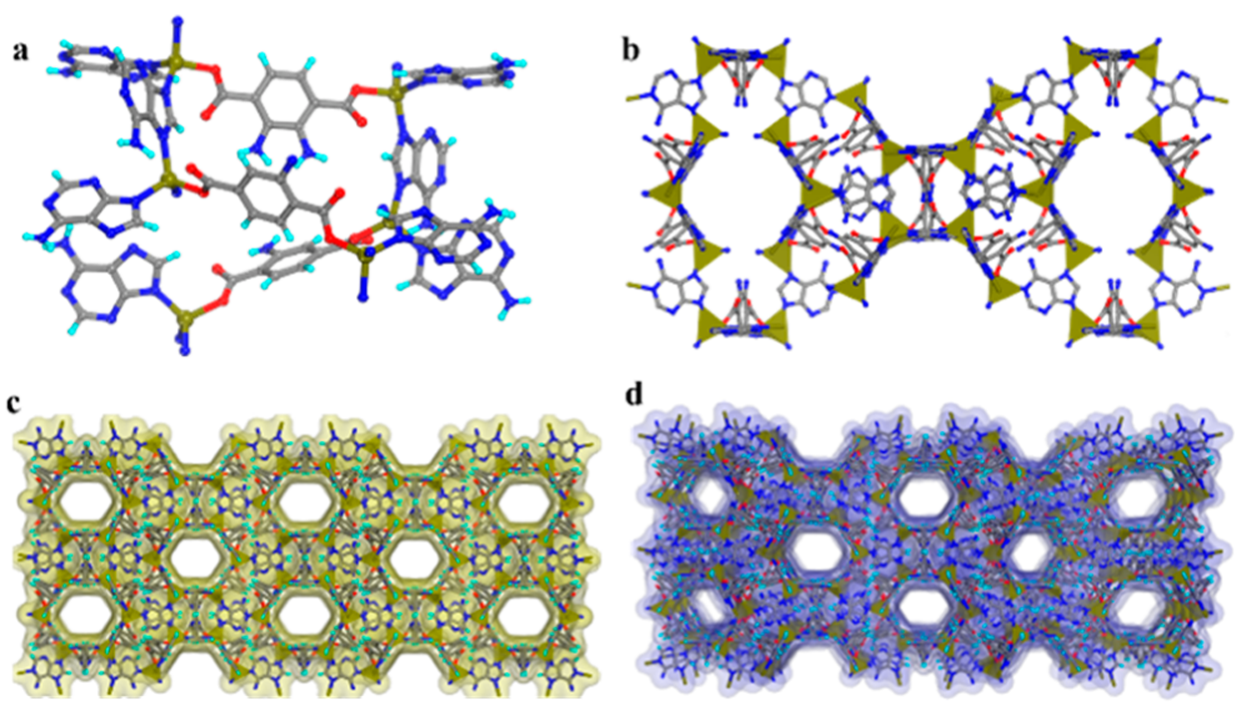
(a) Tetrahedral coordination of Zn^2+^ with three nitrogen atoms coming from three different adenine units. (b) Unit cell of KBM-1 showing tetra-coordinated Zn^2+^ centers. (c) The packing diagram of KBM-1 in the crystallographic *c* axis. (d) Representation of the 3D packing diagram of KBM-1 with the perspective view. The solvent molecules and cations are squeezed by PLATON tools. (Color code: carbon, gray; hydrogen, cyan/omitted for clarity; oxygen, red; nitrogen, blue; zinc/Zn tetrahedra, dark yellow.)

Single-crystal X-ray diffraction (SCXRD) analysis of KBM-1 showed three independents Zn^2+^ ions with full occupancy, one and a half units of NH_2_–BDC^2–^ ion, a total of four molecules of Ade, and one cationic guest in the asymmetric unit (Figure 1). All the Zn^2+^ centers are coordinated in a tetrahedral fashion by one O atom from NH_2_–BDC units and three N atoms from three different Ade units (Figure 2a). More specifically, the coordinated N atoms are all from the imidazolate N atoms of Ade molecules keeping the other N atoms free, similar to the ZIF-8^49^ coordination mode (Figure S1). The counterions occupy the interior channels of the framework, whereas the Watson–Crick face of Ade is pointed toward the surface of the channel from the side arms (Figure 2b). Each of the two independent ligands connects three metal centers at both ends to form 3D coordination networks, as depicted in Figure 2c. During the formation of these 3D networks, large pockets are generated and filled by guest solvent molecules (MeOH and DMF) and dimethylammonium cations (Figure 2d).

The PLATON calculation without removing counterions indicates that this framework affords a porosity of 39% per unit cell volume (Figure S2).^50^ Unlike previously reported Bio-MOFs,^23,51^ two types of coordination are found in the crystal structure of KBM-1 that include the imidazole N atoms while leaving the other N atoms free for hydrogen bonding interactions, as seen in nature for the double-stranded DNA (dsDNA) (Figure S3). We hypothesize that the tetrahedral coordination environment is contributing to this open-face confirmation. In an attempt to understand the supramolecular interaction between the 3D networks, the packing and hydrogen bonding interactions were analyzed in detail. The 3D nets oriented in the *ab* plane are involved in intermolecular N–H···N interactions with the adjacent sheets from either side. Thus, the migrated amine hydrogens H1 of Ade undergoes N–H···N interactions with the Ade nitrogen of the neighboring sheets on either side, generating a hydrogen-bonded Watson–Crick face (Figure S4).

KBM-1 was studied using FT-IR spectroscopy, powder X-ray diffraction (PXRD), and thermal gravimetric analysis (TGA) to assess the thermal/chemical stability and phase purity of the framework. FT-IR analysis revealed the characteristic peaks of Ade and carboxylate. The peaks at 3429 and 3333 cm^–1^ belong to −NH_2_ stretching, and the 3271 cm^–1^ band is assigned to N–H stretching of the heterocyclic ring (Figure S5). The bulk-phase purity was confirmed by comparing the experimental PXRD spectrum with that of the simulated SCXRD data (Figure S6). The preliminary stability of KBM-1 in the open air was verified for one month (Figure S6). It is also stable in water (pH 7) for over 1 month as supported by PXRD results (Figure S7). The chemical stabilities of KBM-1 toward different acidic and alkaline conditions have also been verified (Figure S8). TGA patterns of KBM-1 showed that there is a continuous weight loss with different temperatures; the first weight loss occurs between 30 and 60 °C attributing the removal of the guest molecule MeOH, while the second occurs from 60 to 180 °C is corresponding to the loss of DMF and Me_2_NH_2_^+^. Following the weight loss of the Me_2_NH_2_^+^ cation the framework starts to decompose. (Figure S9). Furthermore, to confirm the thermal stability of these frameworks, a PXRD study was conducted after solvent exchange and outgassing the samples (Figure S10).

To assess the porosity of the framework, N_2_ sorption isotherm of the activated sample displays an uptake of 115.3 cm^3^ g^–1^ at 1 bar pressure with a steep increase in adsorption under lower pressure (Figure S11). The Brunauer–Emmett–Teller (BET) surface area was estimated to be 323 m^2^ g^–1^. It showed a type IV isotherm with a small increase in the uptake at high relative pressure leading to a moderately hysteretic desorption profile. This is in agreement with the surface porosity arising from the apertures between the KBM-1 crystals. The desorption profile also supports the microporosity of the crystalline KBM-1. CO_2_ gas sorption isotherm shows that the uptake capacity is 39 cm^3^ g^–1^ at 298 K and atmospheric pressure; which might be the existence of N-enriched basic sites on to the frameworks which interacts more strongly with CO_2_ gas molecules having a large quadruple moment (Figure S12). The diameter of the KBM-1 channel was calculated as approximately 12.5 × 10.6 Å^2^, taking into account the VDW radius (Figure S13). The particles’ shape and size were characterized by scanning electron microscopy (SEM) and transmission electron microscopy (TEM) and showed a well-defined hexagon-shaped structure with a size range of 100–200 nm (Figures S14 and S15). Moreover, SEM images were collected after incubation in water and media and displayed excellent crystallinity and negligible size change (Figure S16).

**Figure 3:**
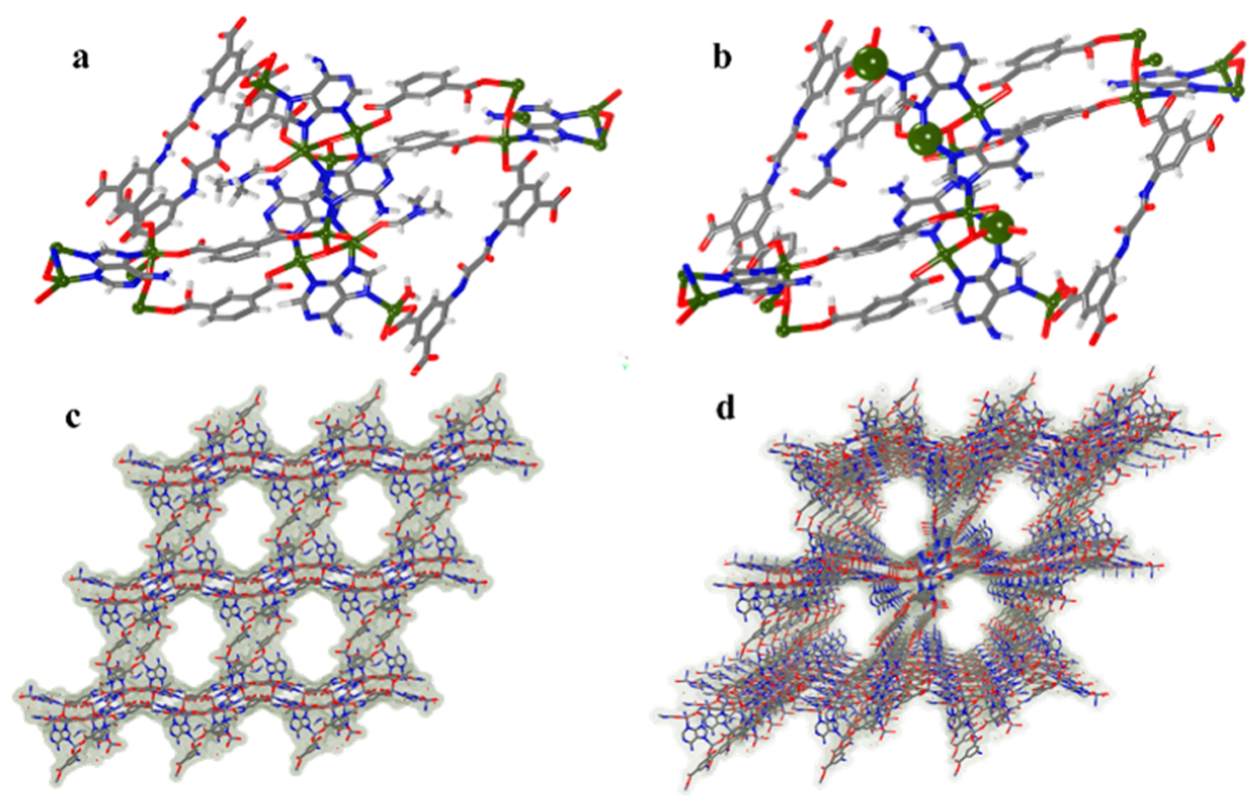
(a) Single-crystal structural information showing the coordination environment of KBM-2. (b) Open Zn^2+^ sites shown as the big green balls. (c) 3D framework along crystallographic *c* axis. (d) Perspective view of the 3D framework. (Color code: carbon, gray; hydrogen, white/omitted for clarity; oxygen, red; nitrogen, blue; Zn, dark yellow.)

SCXRD analysis of KBM-2 showed eight independent Zn^2+^ ions with full occupancy, one full and four half of the L^2–^ linker, and four of the Ade unit and solvent molecules (DMF and H_2_O) in the asymmetric unit (Figure 1). Among the different Zn^2+^ ions one is coordinated by four O atoms from four L linkers and one N atom from the Ade unit at the vertex resulting in a five coordinated square pyramidal geometry (Figure 3a). The other Zn^2+^ center is coordinated in a tetrahedral fashion by two O atoms from two L units and two N atoms from two Ade units. Interestingly, three Zn^2+^ centers are occupied by DMF and water making them susceptible to further complexation upon activation (Figure 3b and Figure S17). The packing diagram indicates that the framework is arranged in a head-to-tail fashion to create the interconnected layered structures along the *a* axis (Figure 3c). The 3D nets oriented in the *bc* plane generate free Watson–Crick faces that are pointing toward the channels of KBM-2 (Figure 3d). The pore size of the KBM-2 channel was calculated as approximately 16.7 × 5.6 Å^2^, taking into account the VDW radius (Figure S18). N_2_ sorption isotherm of the activated sample showed an uptake of 141.5 cm^3^ g^–1^ at atmosphereic pressure with a BET surface area of 407 m^2^ g^–1^ (Figure S19). The PLATON calculation indicates that this framework affords a porosity of 51% unit cell volume. TGA analysis of KBM-2 revealed a weight loss of 17% below 100 °C, which is attributed to the loss of lattice solvents, whereas the framework is thermally stable until 250 °C (Figure S20). The phase purity of the bulk material was established by PXRD study, which revealed a good agreement with the corresponding simulated patterns obtained from the single-crystal data (Figure S21). To assess its water stability, we immersed KBM-2 in filtered water and dried it. The PXRD pattern of the dried sample was the same as the as-synthesized pattern (Figure S21). Moreover, we have tested the framework sustainability of KBM-2 in different pH (Figure S22). FT-IR analysis revealed the characteristic peaks of Ade and carboxylate. The peaks at 3445 and 3299 cm^–1^ belong to −NH_2_ stretching, and the 1694 cm^–1^ band is assigned to the −C=O stretching of the heterocyclic ring (Figure S23). The particles shape and size were characterized by SEM and TEM and showed a needle-shaped structure with a size range of 500–600 nm (Figures S24 and S25). Further SEM studies were also conducted after incubation in water and media (Figure S26).

The accessibility of the Watson–Crick face of Ade in both KBM-1 and KBM-2, as supported by SCXRD, encouraged us to study the possible hydrogen bonding interactions with guest molecules such as Thy through base-pairing. Thy loading experiments were performed in a purely aqueous solution with loadings ranging from 30 to 100%. Attempts to obtain SCXRD data of the loaded frameworks were unsuccessful; however, both systems retained their crystallinity as supported by PXRD data (Figure S27). Consequently, a detailed theoretical study was performed using the self-consistent charge density functional tight-binding (SCC-DFTB) method to optimize the simple geometries of KBM-1 and KBM-2. As depicted in Figures S28 and S29, hydrogen bonding and van der Waals type interactions with two Thy molecules can be seen. A blue isosurface representing hydrogen bonding is easily recognized between Thy and Ade molecules (Figure S29). Further experimentation was carried out to better verify the Thy molecules interaction with the frameworks starting with BET where the surface area of KBM-1 drastically decreased by 295 m^2^ g^–1^ after treatment with Thy (Figure S30). FT-IR spectra of Thy@KBM-1 and Thy@KBM-2 showed an additional peak related to the carbonyl group at 1706 and 1693 cm^–1^, respectively which is characteristic stretching vibration peak positions of Thy molecule (Figures S23 and S31). In addition, we have cautiously collected the solid state CP/MAS NMR spectra ^1^H and ^13^C for Thy@KBM-1 and Thy@KBM-2 and their parent materials confirming the purity and the structural stability in all cases. ^13^C solid-state NMR (CP/MAS) spectra showed shifts in Thy@KBM-1 at δ 152.52 ppm and a new peak at δ 14.45 ppm confirms the diffusion of Thy within the pores of KBM-1 (Figures S32–S34). Moreover, ^1^H solid state NMR spectra further confirmed the shifts, and intensity changes in both the spectra of Thy@KBM-1 and Thy@KBM-2 compared to the guest-free frameworks. This further discloses the insertion of Thy within the pores of KBM-1 and KBM-2 via complementary hydrogen bonding and van der Waals type interactions (Figures S35–S38).

**Figure 4:**
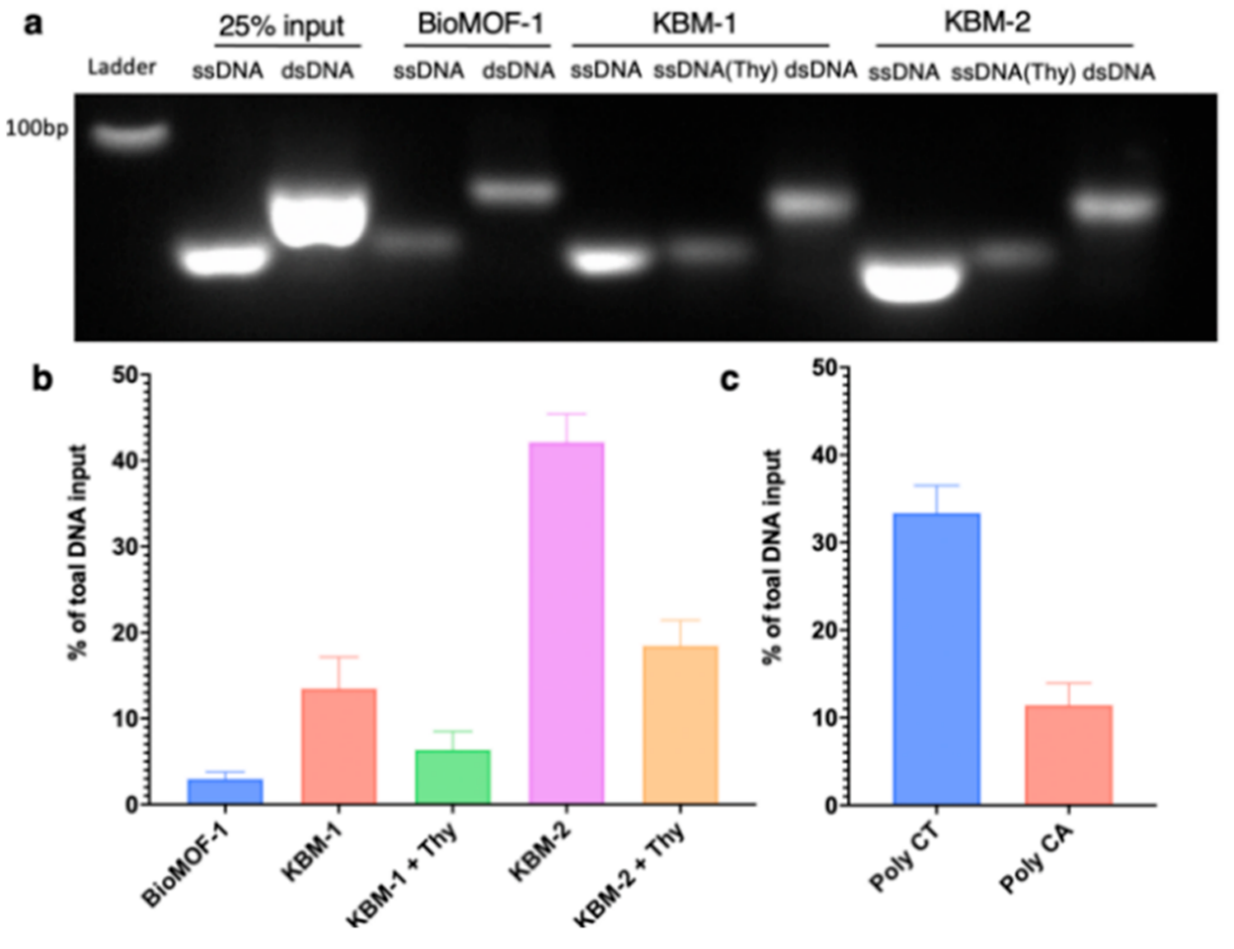
Agarose gel electrophoresis analysis of (a) ssDNA and dsDNA loading on BioMOF-1, KBM-1, and KBM-2 in the presence and absence of free Thy (as a competitive guest). Fluorescence-based quantitative analysis of ssDNA: (b) 49-mer oligonucleotides ssDNA loading on BoiMOF-1, KBM-1, and KBM-2 in the presence and absence of free Thy (*n* = 3), (c) ssDNA poly CT and poly CA loading on KBM-2 (*n* = 3).

**Figure 5:**
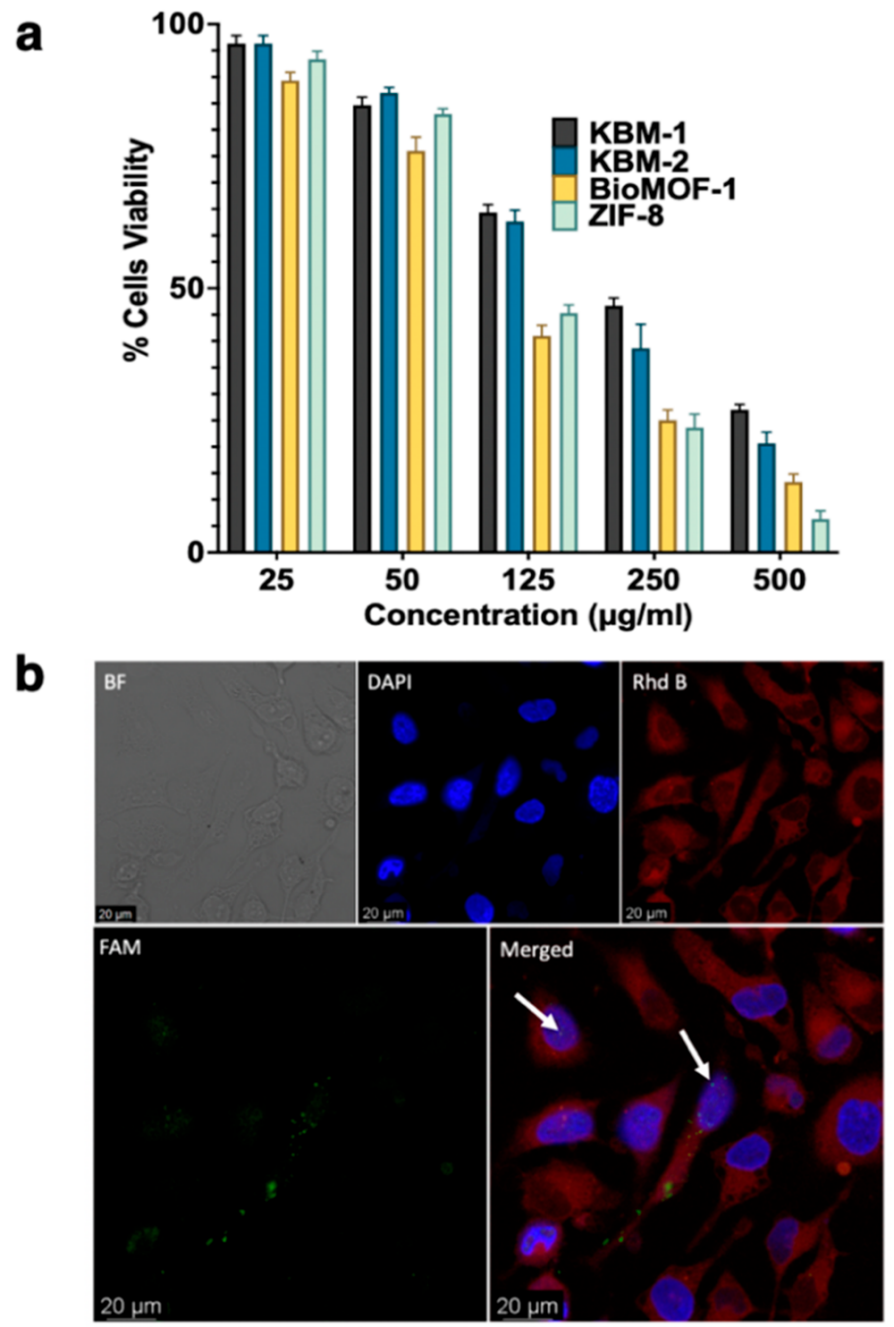
(a) Cytotoxicity evaluation of KBM-1, KBM-2, BioMOF-1, and ZIF-8 using an MTT assay (*n* = 3). (b) Analysis of ssDNA@KBM-2 cellular uptake and ssDNA delivery using confocal microscopy. The arrows point to ssDNA anti-PCNA in the nucleus.

We then ventured to check the applicability of KBM-1 and KBM-2 in mimicking natural DNA base pairing interactions by using single-stranded DNA (ssDNA) as cargo molecules. We also tested the same sequence but with double-stranded DNA (dsDNA), as a control and prepared the well-known BioMOF-1 to compare its viability and performance with both KBM-1 and KBM-2. First, 49-nucleotides ssDNA and dsDNA (10 μM) were individually mixed with a buffer solution (20 mM Tris-HCl pH 7) of BioMOF-1, KBM-1, KBM-2, and KBMs pretreated with Thy (Figure 4). Gel electrophoresis analysis showed that KBM-2 has superior retention of ssDNA followed by KBM-1 then BioMOF-1. In addition, a reduction in the ssDNA loading was realized when KBM-1 and KBM-2 were pretreated with free Thy (Figure 4a). Employing dsDNA as the genetic cargo also showed a decrease in the loading capacity of both KBM-1 and KBM-2. We rationalize that the loading is happening on the Ade open face of the framework rather than as a typical DNA encapsulation. An ssDNA quantitative assay showed that KBM-2 loaded 41% of ssDNA compared to 13% for KBM-1 (Figure 4b). To further verify the role of base-pairing, we compared the loading of two DNAs, Thy-rich poly CT and Thy-free Poly CA oligonucleotides, on KBM-2. ssDNA quantitative assay analysis indicated that KBM-2 loaded Thy-rich ssDNA (Poly CT) more efficiently than Poly CA (Figure 4c). To compare our system with ZIF-8, we have analyzed ssDNA loading and showed that ZIF-8 has a 55% loading efficiency, which is comparable to that of KBM-2 (Figure S39). However, the difference in biocompatibility favors KBM-2 for ssDNA delivery applications (Figure 5a). Ultimately, although different types of interactions are involved in the ssDNA loading on the framework, complementary base pairing plays an important role in promoting this loading. We believe that ssDNA moieties are located primarily on the surface of the MOFs through several noncovalent interactions, as supported by the computational study with Thy@KBM-1 and Thy@KBM-2 (Figures S28 and S29). After careful evaluation of both of the structures, we realized that KBM-2 is a neutral framework, unlike KBM-1, which is anionic in nature. Moreover, the totally exposed Watson–Crick sites in the case of KBM-2 is more favorable than that of KBM-1; approving KBM-2 to load more ssDNA than that of KBM-1. On the other sites, it is well-known that the defects in the crystal structure favors the loading capacity toward guests.

To investigate the applicability of KBM-1 and KBM-2 for prospective biomedical applications, we analyzed their biocompatibility, pH-triggered cargo release, and eventual cellular uptake. The cytotoxicity of KBM-1 and KBM-2 was established by the MTT assay (three replicates) and compared to the commonly used ZIF-8 and BioMOF-1. KBM-1 and KBM-2 showed superior biocompatibility with IC_50_ values of 190 and 155 μg/mL, respectively, compared to 112 μg/mL for Bio-MOF-1 and 95 μg/mL for ZIF-8 (Figure 5). A similar cytotoxicity profile was concluded for both cancer (HeLa) and normal (human dermal fibroblast) cells (Figure S40). Moreover, KBM-1 and KBM-2 can protect the loaded ssDNA from digestion by DNase I, a nuclease that is responsible for the degradation of the majority of circulating DNA derivatives. The digestion test was reproducibly repeated, where the ssDNA@KBMs samples showed excellent stability even after 30 min of incubation with DNase I (Figure S41). Furthermore, the release profiles of ssDNA from KBM-1 and KBM-2 in response to pH change have been investigated where a significant release was noted at pH 5.5 mainly due to the degradation of the framework (Figure S42). Control studies using PXRD were performed for KBM-1 and KBM-2 in H_2_O, PBS (pH 7) and media (Figure S43). Finally, a dynamic light scattering (DLS) study in aqueous media was conducted to verify the colloidal stability of KBM-1 and KBM-2 (Figure S44). Considering the obtained data, we can directly deduce that the overall behavior of these frameworks fits very well with the behavior of ZIFs in acidic PBS.

As KBM-2 has the highest loading capacity compared to KBM-1, we analyzed its cellular uptake and consequent delivery of loaded ssDNA in HeLa cells. To visualize KBM-2, we stained it with Rhodamine B (Rhd B) fluorophore and loaded ssDNA (Anti-PCNA) labeled with fluorescein amidites (FAM) fluorophore. Anti-PCNA is a DNA replication inhibitor and can promote cell death.^39,40^ HeLa cells were then incubated with a control, KBM-2, or ssDNA@KBM-2 for 4 h (Figure S45). Confocal microscopy analysis showed that both FAM and Rhd B fluorescence were observed inside the cells when they were treated with ssDNA@KBM-2 (Figure 5b and Figure S45). Furthermore, the green FAM fluorescence of ssDNA was observed in the cell nucleus (Figure 5b). No cellular uptake was observed when the cells were treated with anti-PCNA alone (Figure S46). The anti-PCNA activity was verified by the ability of anti-PCNA@KBM-2 to inhibit cell growth and promote cell death compared to KBM-2 and nonfunctional ssDNA (control ssDNA@KBM-2), whereas no significant toxicity was detected using ssDNA oligomers alone (Figure S47 and S48).

## Conclusion

In conclusion, two biomimetic 3D porous frameworks KBM-1 and KBM-2 are reported herein with excellent stability and biocompatibility. Similar to BioMOF-1, both KBM-1 and KBM-2 are adenine (Ade)-based; however, an unnatural amino acid linker and amide functional group have been used to free the adenine coordination sites, which brings it closer to the Watson–Crick model with an accessible Ade face. KBM-1 and KBM-2 can significantly load 13% and 41% of ssDNA, respectively. Moreover, the role of Ade-Thy base pairing in promoting ssDNA loading was successfully verified using Thy competitive-guest binding and theoretical calculations. The enhanced biocompatibility and the aqueous stability empower this class of Bio-MOFs with impressive potential to be eventually translated into smart biomedical platforms, especially for genetic material stabilization, storage, and delivery.

## Experimental Section

### Synthesis of KBM-1

A mixture of 59 mg of Zn(NO_3_)_2_·6H_2_O (0.2 mmol), 27.4 mg of adenine (0.2 mmol), 18.8 mg of 2-amino-1,4-benzene dicarboxylic acid (0.1 mmol), 3 mL of *N*,*N*′-dimethylformamide (DMF), 2 mL of MeOH, and 3 mL of water were added into a 15 mL scintillation vial followed by 30 min sonication to obtain a clear solution. The scintillation vial was then sealed into a stainless steel vessel and heated at 110 °C for 48 h followed by cooling down to room temperature at a rate of 5 °C/h. Pale yellow rectangular crystals of KBM-1 suitable for single-crystal X-ray diffraction were collected by filtration. The crystals were rinsed with fresh DMF, MeOH, and H_2_O (2 × 20 mL) and dried in air, giving a yield of 45%. Elemental analysis calcd (%) for C_37_H_38_N_24_O_7_Zn_3_: C 39.43, H 3.40, N 29.83; found: C, 40.05, H 3.49, N 30.09; ICP-OES; amount of Zn(II): 17.81% with respect to the exact amount (17.41%).

### Synthesis of KBM-2

A mixture of 59 mg of Zn(NO_3_)_2_·6H_2_O (0.2 mmol), 27.4 mg of adenine (0.2 mmol), 34.4 mg of **L**(52) (0.1 mmol), 3 mL of *N*,*N*′-dimethylformamide (DMF), 2 mL of water with 4 drops of conc. HNO_3_ were added into a 15 mL scintillation vial followed by 30 min of sonication to obtain a clear solution. The scintillation vial was then sealed into a stainless steel vessel and heated at 110 °C for 48 h followed by cooling down to room temperature at a rate of 5 °C/h. Colorless needlelike crystals of KBM-2 suitable for single-crystal X-ray diffraction were collected by filtration. The crystals were rinsed with fresh DMF, MeOH, and H_2_O (2 × 20 mL) and dried in air, giving a yield of 63%. Elemental analysis calcd (%) for C_82_H_69_N_26_O_37_Zn_8_: C 38.87, H 2.74, N 14.37; found: C, 38.98, H 2.89, N 15.02; ICP-OES; amount of Zn(II): 21.03% with respect to the exact amount (20.65%).

## Supporting Information Available

Characterization, including figures and tables, SCXRD data, crystallographic figure and parameters, PXRD, TGA, and details of the bio study (PDF)
