# Supplementary material for: DNA-Mimicking Metal–Organic Frameworks with Accessible Adenine Faces for Complementary Base Pairing
Source: JACS Au. 2022 Feb 7;2(3):623–30. doi: 10.1021/jacsau.1c00516 (PMC8969998; doi:10.1021/jacsau.1c00516)
Supplement: Supplementary file 1 — au1c00516_si_001.pdf [file au1c00516_si_001.pdf]

Supporting Information

DNA-Mimicking Metal-Organic Frameworks with Accessible Adenine Faces for Complementary Base Pairing

Santanu Chand,[1 ⊥] Othman Alahmed,[1 ⊥] Walaa S. Baslyman,[1] Avishek Dey,[1] Somayah Qutub,[1] Ranajit Saha,[2] Yuh Hijikata,[2] Manal Alaamery,[3] Niveen M. Khashab[1*]

[1] Smart Hybrid Materials (SHMs) Laboratory, Advanced Membranes and Porous Materials Center, King Abdullah University of Science and Technology (KAUST), Thuwal 23955-6900, Kingdom of Saudi Arabia

[2] Institute for Chemical Reaction Design and Discovery (WPI-ICReDD), Hokkaido University, Sapporo, Hokkaido 001-0021, Japan

[3] Developmental Medicine Department, King Abdullah International Medical Research Center, King Saud Bin Abdulaziz University for Health Sciences, Ministry of National Guard- Health Affairs (MNG-HA), Riyadh 11481, Saudi Arabia

Experimental Section

Materials. All reagents and solvents were purchased from commercial sources and used without further purification. Linker **L** has been synthesized from previously reported procedure.[1]

Physical Measurements. The Fourier transform infrared (FTIR) spectra were recorded using a Thermo Scientific spectrometer (Nicolet iS10). PXRD patterns were recorded using Cu-Kα radiation (1.5418 Å) on a Bruker D8 Advance diffractometer. Thermogravimetric analysis (TGA) measurements were carried out on a Q5000 (TA Instruments) and the samples were heated from room temperature to 600 °C at a rate of 5 °C min^{-1} under N_2 atmosphere. Low-pressure gas adsorption measurements were performed using a Microactive 4.0 ASAP 2020 instrument. To remove all guest solvents in the framework, the fresh samples were first solvent-exchanged with dry acetone at least 10 times within 2 days and degassed at 343 K for 12 h. The sorption measurement was maintained at 77 K under liquid nitrogen. Room temperature CO_2 Gas sorption experiments were tested on a Micromeritics ASAP 2020 instrument. Solid state NMR spectra were acquired on an Avance III 600 WB spectrometer equipped with a 3.2 mm low-temperature CPMAS probe capable of spinning speed up to 24 kHz. Transmission electron microscopy (TEM) was conducted on FEI Titan-ST electron microscope. Dynamic light scattering (DLS) and zeta potential analysis were performed using a Malvern Nano ZS instrument at 25 °C.

Single Crystal X-ray Diffraction. The crystal and refinement data for ***KBM-1*** and **2** were collected in Table S1 and Table S2. In this case, a crystal of suitable size was selected from the mother liquor and immersed in paratone oil and then it was mounted on the tip of glass fiber and cemented using epoxy resin. Single crystal X-ray data were collected at 120 K on a Bruker SMART APEX II CCD diffractometer using graphite-monochromated Mo-K_α radiation (0.71073 Å). The linear absorption coefficients, scattering factors for the atoms and the anomalous dispersion corrections were taken from International Tables for X-ray Crystallography. The data integration and reduction were processed with SAINT

software.[2] An empirical absorption correction was applied to the collected reflections with SADABS using XPREP.[3] The structure was solved by the direct method using SHELXTL and was refined on F^2 by a full-matrix least-squares technique using the SHELXL-2014 program package.[4-6] For all the cases, non-hydrogen atoms were refined anisotropically. Attempts to identify the highly disordered solvent molecules were failed. Instead, a new set of F^2 (hkl) values with the contribution from the solvent molecules withdrawn were obtained by the SQUEEZE[7] procedure implemented in PLATON.[8] The cationic guests $Me_2NH_2^+$ could be located in the channels from Difference Fourier Map for **KBM-1**.

Computational Details: The initial structure of the **KBM-1** and **KBM-2** are prepared using the experimentally obtained crystal structures. non-coordinating solvent DMF molecules are removed from the **KBM-1** model, whereas DMF and H_2O molecules coordinating to Zn were left in the **KBM-2** model. The geometries of **KBM-1** and **KBM-2** were optimized using self-consistent-charge density-functional tight-binding (DFTB) method[9] with the third-order expansion[10] as implemented in the DFTB+ package version 18.2.[11] The 3ob parameter set with Hubbard parameters, -0.1492 for C, -0.1857 for H, -0.1575 for O, -0.03 for Zn and -0.1535 for N were used,[12] and Grimme's D3 type dispersion was included.[13] Since the MOF is an infinite system, periodic boundary condition was applied for the both frameworks, and the cell parameters were fixed to the experimental values during the optimizations. After two Thy are introduced into the pore channels of **KBM-1** and **KBM-2**, we again optimized Thy@**KBM-1** and Thy@**KBM-2** with the fixed cell parameter as well.

To shed light on the noncovalent interactions (NCI) between the Thy and **KBM** frameworks of Thy@**KBM-1** and Thy@**KBM-2**, NCIPLOT software was used.[14-15] The relation between the electron density (ρ) and its reduced density gradient (s) is as follows,

$$s = \frac{1}{2(3\pi^2)^{1/3}} \frac{|\nabla\rho|}{\rho^{4/3}},$$

where $\nabla\rho$ is the gradient of ρ. The promolecular approach has been considered for the computations in this work. The sign(λ_2)ρ as the reduced density gradient (s) is plotted using VMD visualization package.[16]

***MTT assay for cytotoxicity evaluation*:** HeLa and HDF cells were seeded in a 96-well plate at 1X10^4 cells/well. Cells were incubated at 37 °C, 5% CO_2 for 24 hours, then different concentrations of **KBM-1**, **KBM-2,** BioMOF-1[17] and ZIF-8[18] were added to cells. Cells in media containing MOFs were incubated for 24 hours. Then, the MTT assay was performed following manufacturer protocol (BioVision #K299-1000). Three biological replicates were analyzed using Bio-Rad XMark microplate spectrophotometer. Same procedure was used to evaluate control ssDNA, anti-PCNA aptamer, **KBM-2**, ssDNA **KBM-2**, control ssDNA@**KBM-2** and Anti-PCNA@**KBM-2**. The optimal concentration of free ssDNA control and Anti-PCNA was calculated based on **KBM-2** ssDNA loading efficiency (~ 41%) as follows:
Amount of DNA to be added to cell (µg) = (DNA in loading reaction in µg X 0.41)/ (5000/ corresponding **KBM-2** in µg)

KBMs ssDNA loading: 5mg of **KBM-1**, Thy@**KBM-1**, **KBM-2**, Thy@ **KBM-2** or BioMOF-1 were prepared in 250 µl of 20mM Tris-HCl pH 7 and 10 µM of DNA, were mixed. The mixture was incubated for 4 hours at 25 °C, 900 RPM. Then BioMOFs were centrifuged at 3000 RPM For 10 mins. Supernatants were collected for analysis of unloaded DNA. KBMs were washed twice with the same buffer and vacuum dried.
KBMs were resuspended in 250 µl of 20 mM Tris-HCl pH 7 buffer and boiled at 95 °C for 5 mins. DNA retention of KBMs was analyzed by loading 20µl of Boiled KBMs and 5µl of input (10µM DNA in 250µl 20mM Tris-HCl pH 7) to 2% agarose gel electrophoresis. Agarose gel was subjected to electrophoresis at 80 V for 90 mins. In addition, we analyze the percentage of unloaded DNA in supernatant of loading reaction using ssDNA and dsDNA quantitative assays (Thermofisher scientific # O11492 and Q33120) following the manufacturer protocol. Percentage of Loaded DNA was calculated as following 100-% unloaded= % loaded DNA.

Loaded oligonucleotides
*Control ssDNA and DsDNA (5’-
ATCGATCGATCGATCGATCGATCGATCGATCGATCGATCGAT CGATCGA-3’)

* Poly CT (5’-CTCTCTCTCTCTCTCTCTCTCTCTCTCTCTCTCTCTCTCTCTCTCTCTCTC-3’).
* Poly CA (5’-CACACACACACACACACACACACACACACACACACACACACACACACACAC-3’).
* Anti-PCNA Aptamer (5’-CATGCTTCCCCAGGGAGATGCCTATGGTCCCCGCGTAGG TGGCAGCTCA -3’)[19]

ssDNA release analysis. **KBM**s loaded with ssDNA labeled with Fluorescein amidite (FAM) were redispersed in 250µl of 20 mM Tris-HCl at either pH-7 or pH-5.5 incubated at 25 °C, 900 rpm. Release profiles were analyzed by quantifying the FAM signal in the release buffer over 24hours. We quantify FAM form release buffer collected at time points (0.5, 1, 2, 4, 6, 12, 24 hours) using Tecan plate reader. Intensity of time point were the divided by 41% of input intensity in case of **KBM-2** and 13% for **KBM-1**.

***DNase I digestion Reaction*:** To normalize ssDNA input, we added amounts of **KBM-1** and **KBM-2** that load the same amount of DNA as we have in the free DNA control. 1µM of free ssDNA, 3.8mg of ssDNA@**KBM-1** (5mg loads 10 µM X 13% = 1.3 µM) and 1.2 mg ssDNA@**KBM-2** (5 mg loads 10 µM X 41% = 4.1 µM) were resuspended in 100 ul of DNase I buffer (10 mM Tris-HCl 2.5 mM $MgCl_2$ 0.5 mM $CaCl_2$ pH 7 @37 °C) and divided into two tubes. One tube was kept as a control for DNase I independent degradation and 1 µl of 1U/µl DNase I (Thermo scientific # K0441). Digestion reaction was performed for 30 mins and then enzyme was deactivated by heating at 70 °C for 5 mins. 20 µl of the reaction was analyzed on 2% Agarose gel.

Cellular uptake of KMB-2 and ssDNA KBM-2

$5X10^4$ HeLa cells were seeded in 4-well slide. Cells were left to grow for overnight. Cells were treated with PBS (control), Rhodamine B **KBM-2** (100µg/ ml) and FAM-ssDNA@ Rhodamine B **KBM-2** (100µg/ ml), free FAM labelled anti-PCNA (0.319 µg). Cell were washed and fix with 4% PFA after 4 hours. Cells were then stained with DAPI and imaged using Leica TCS LSI confocal microscope at KAUST core lab.

Table S1. Crystal Data and structure refinements for ***KBM-1***

**Empirical formula**	$C_{37}H_{38}N_{24}O_7Zn_3$
**Formula weight**	1127.02
**Temperature (K)**	120(1)
**Radiation**	Mo-Kα
**Wavelength(λ)**	0.71069 Å
**Crystal system**	orthorhombic
**Space group**	*Pbcn*
***a*[Å]**	29.0608(7)
***b*[Å]**	14.9493(3)
***c*[Å]**	23.5731(5)
α[°]=β[°]=γ[°]	90.00
**Volume[$Å^3$]**	10241.1(4)
Z	8
**Density (calculated) [Mg/m^3]**	1.462
**Absorption coefficient [mm^{-1}]**	1.463
**F(000)**	4592
**Refl. used [I > 2σ(I)]**	11119
**Independent reflections**	15644
**R_{int}**	0.1066
**Refinement method**	full-matrix least squares on F^2
**GOF**	1.069
**Final *R* indices[*I>2σ(I)]***	R_1=0.0499; wR_2=0.1484
***R* indices (all data)**	R_1=0.0746; wR_2=0.1591

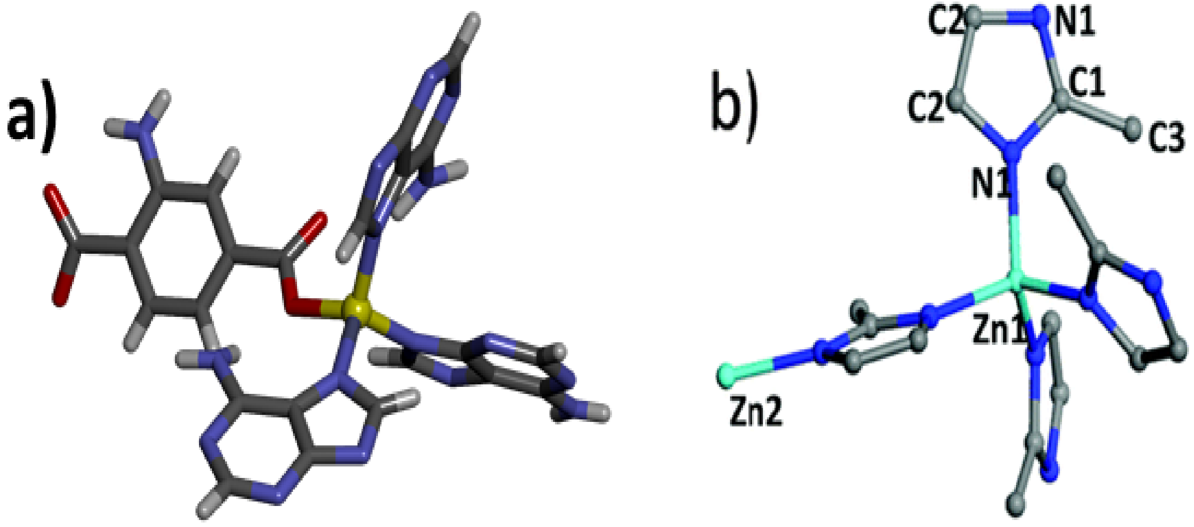

Figure S1. Comparison in the coordination environment between **KBM-1**(a) and ZIF-8 (b) (Color code; Carbon: gray, hydrogen: white, oxygen: red, nitrogen: blue, zinc: dark yellow).

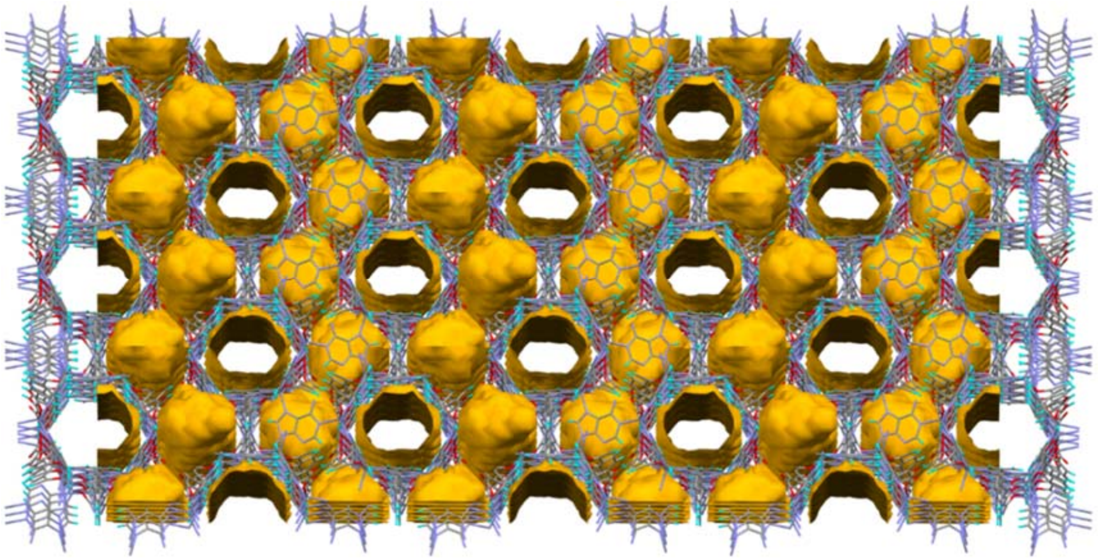

Figure S2. Representation of the packing diagram of **KBM-1** having the solvent accessible volume around 39% of the total volume. The cations and guest solvent molecules are omitted for clarity.

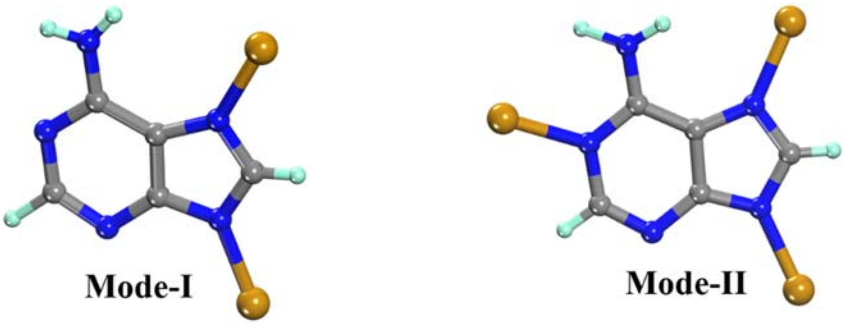

Figure S3. Two types of binding environment in the crystal structure of **KBM-1**.

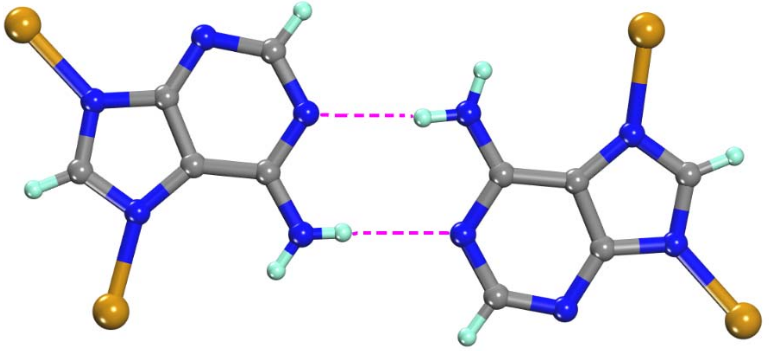

Figure S4. Due to having the unique coordination environment between Zn and Adenine unit, the H-bonding interaction resemblance the *Watson-Crick* model in DNA molecule.

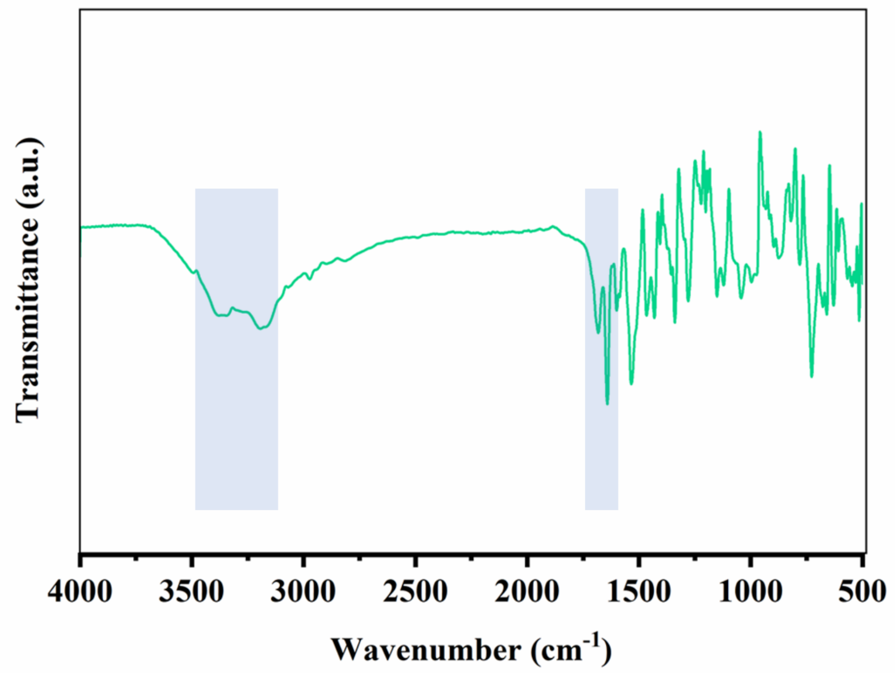

Figure S5. FT-IR spectra of the as synthesized **KBM-1**.

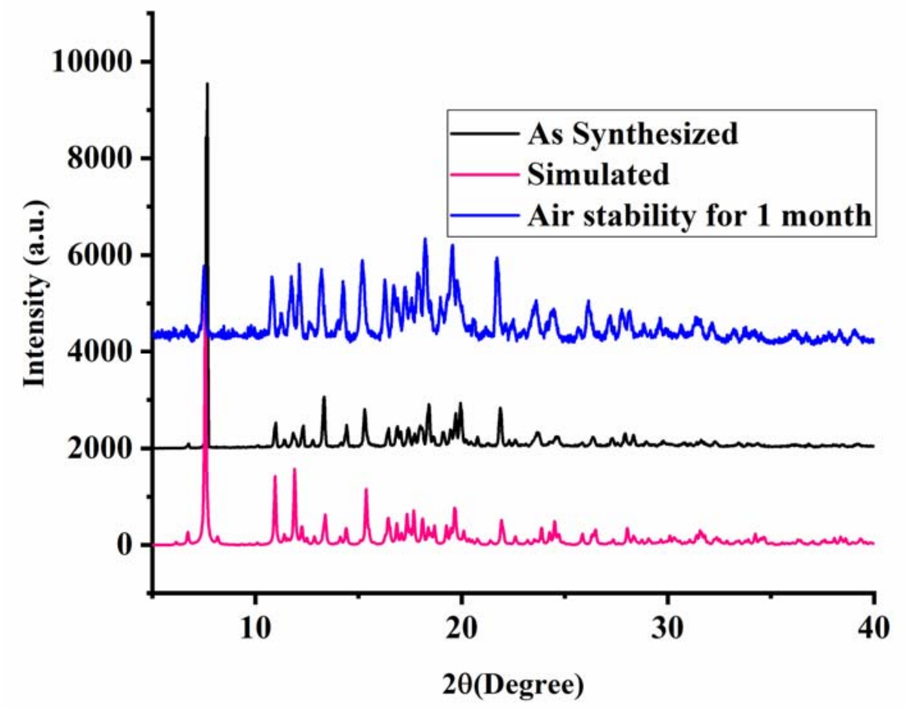

Figure S6. Comparison of the PXRD pattern of **KBM-1** as synthesized (black) with simulated version (pink) and its air stability for 1 month (blue).

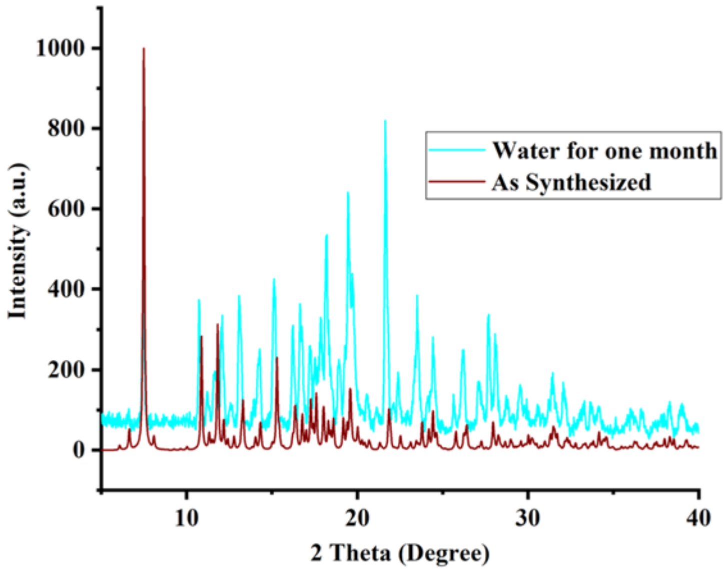

Figure S7. PXRD patterns of **KBM-1**: experimental and after immersion in water for one month.

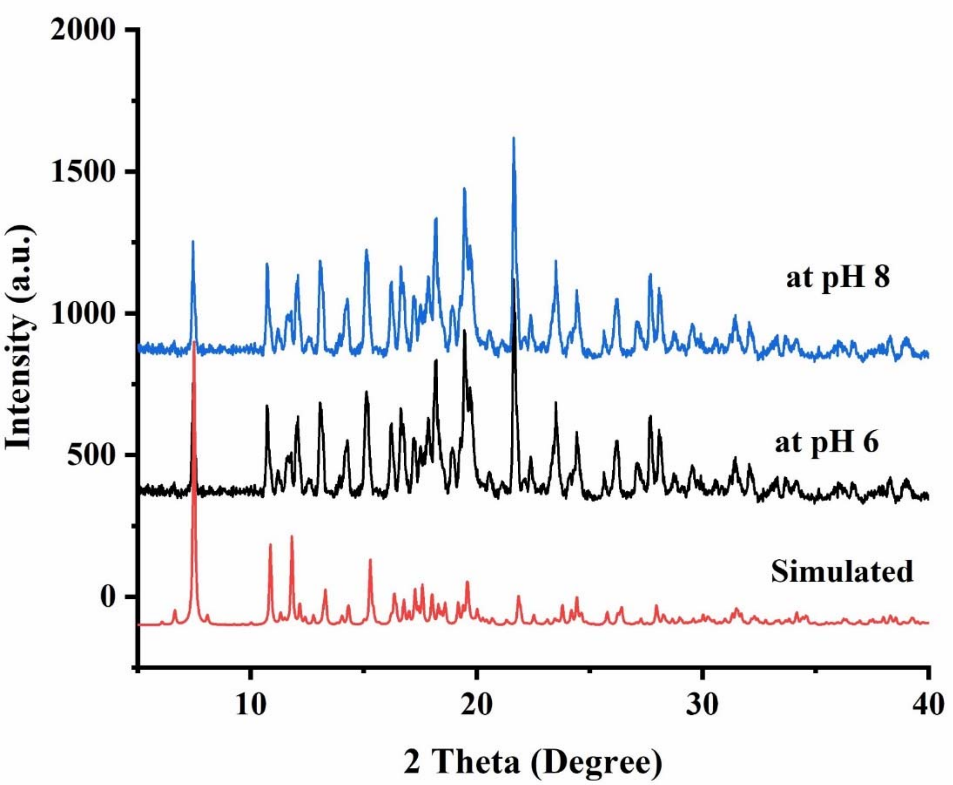

Figure S8. PXRD patterns of **KBM-1**: chemical stability test towards acidic and basic conditions for 24 hours. PBS concentration = 10mM, pH adjusted with HCl.

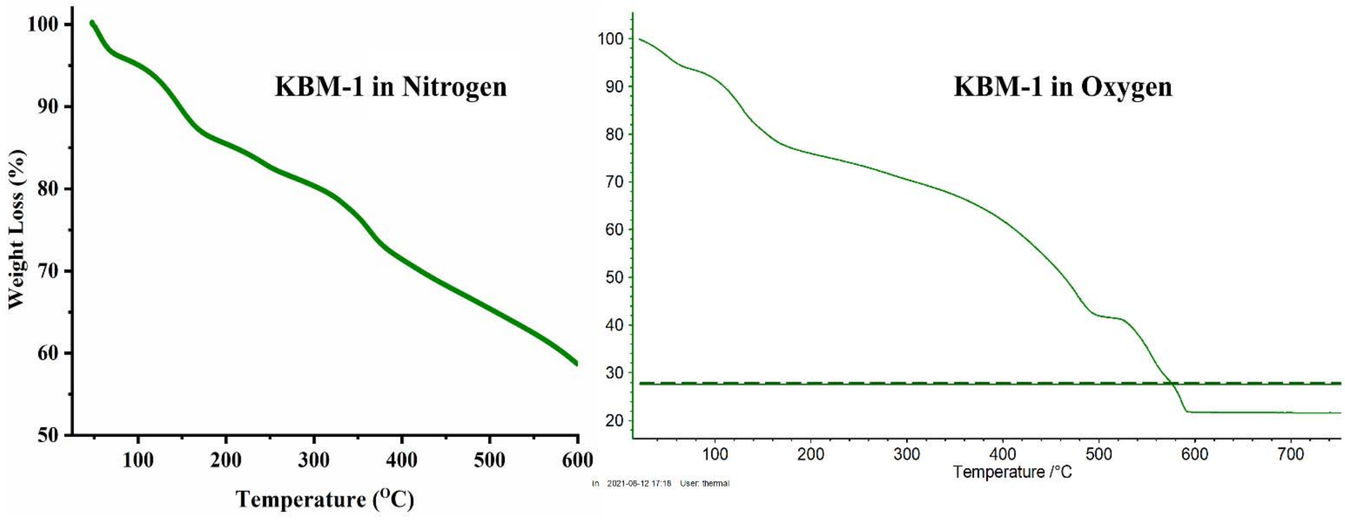

Figure S9. Thermo gravimetric analysis (TGA) data of the as synthesized **KBM-1**.

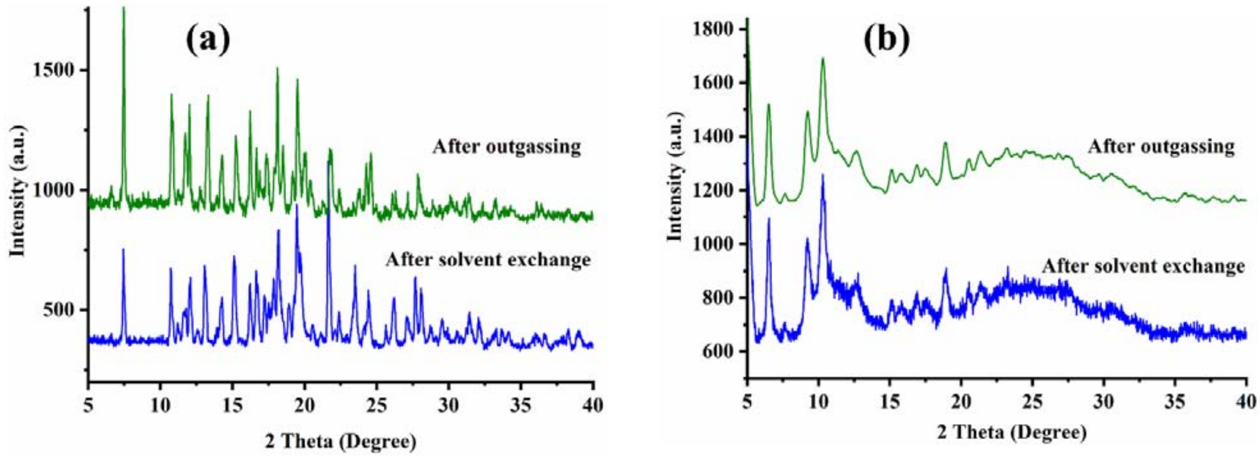

Figure S10. PXRD patterns of **KBM-1** (a) and **KBM-2** (b) after solvent exchange and degassing.

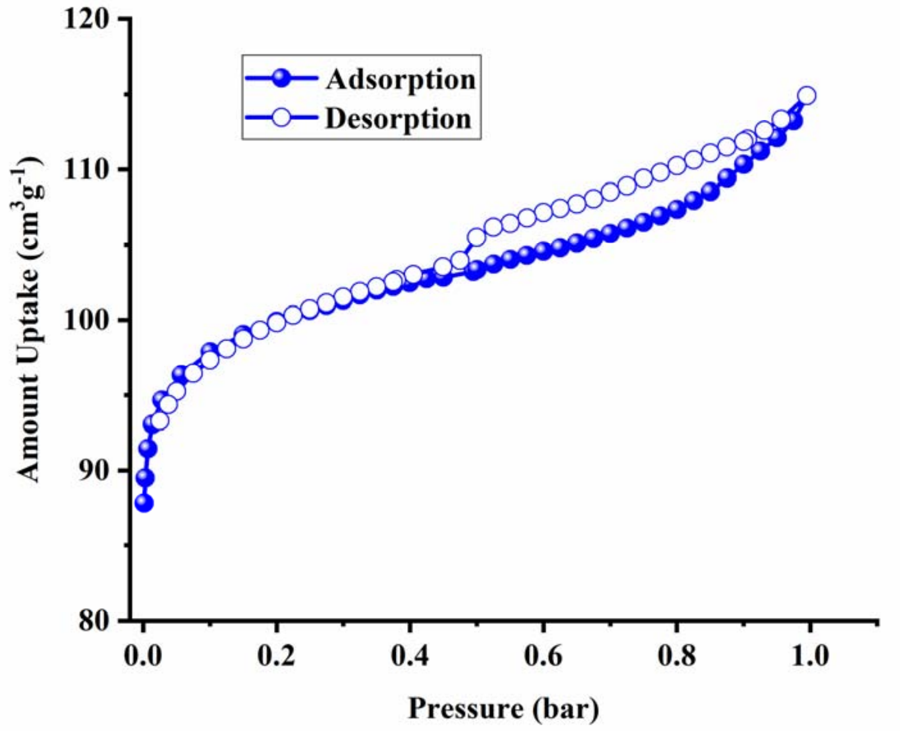

Figure S11. N_2 sorption at 77 K/1 bar of **KBM-1**.

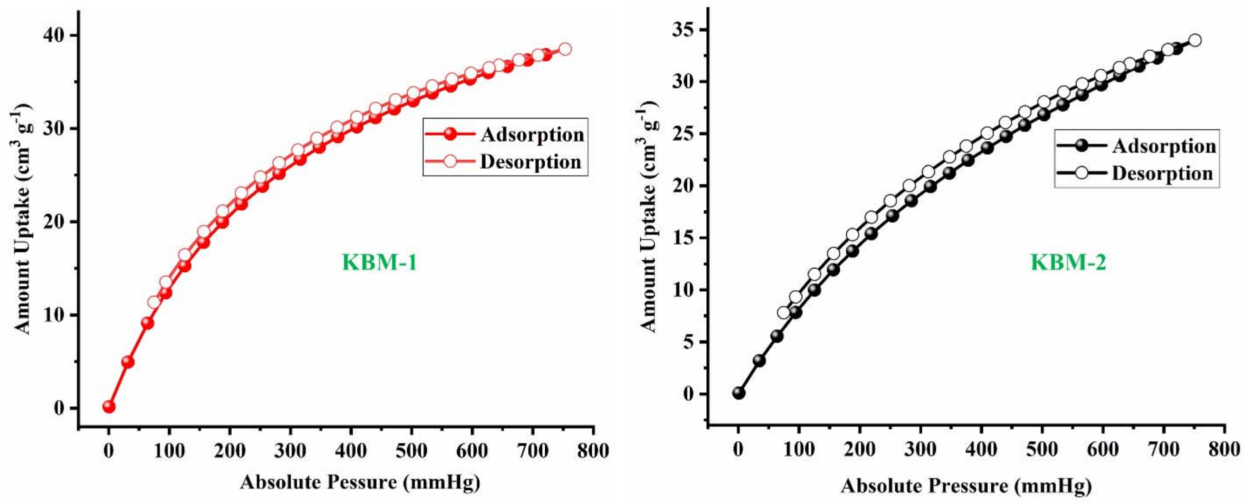

Figure S12. The CO_2 sorption isotherm of **KBM-1** and **KBM-2** at 298 K.

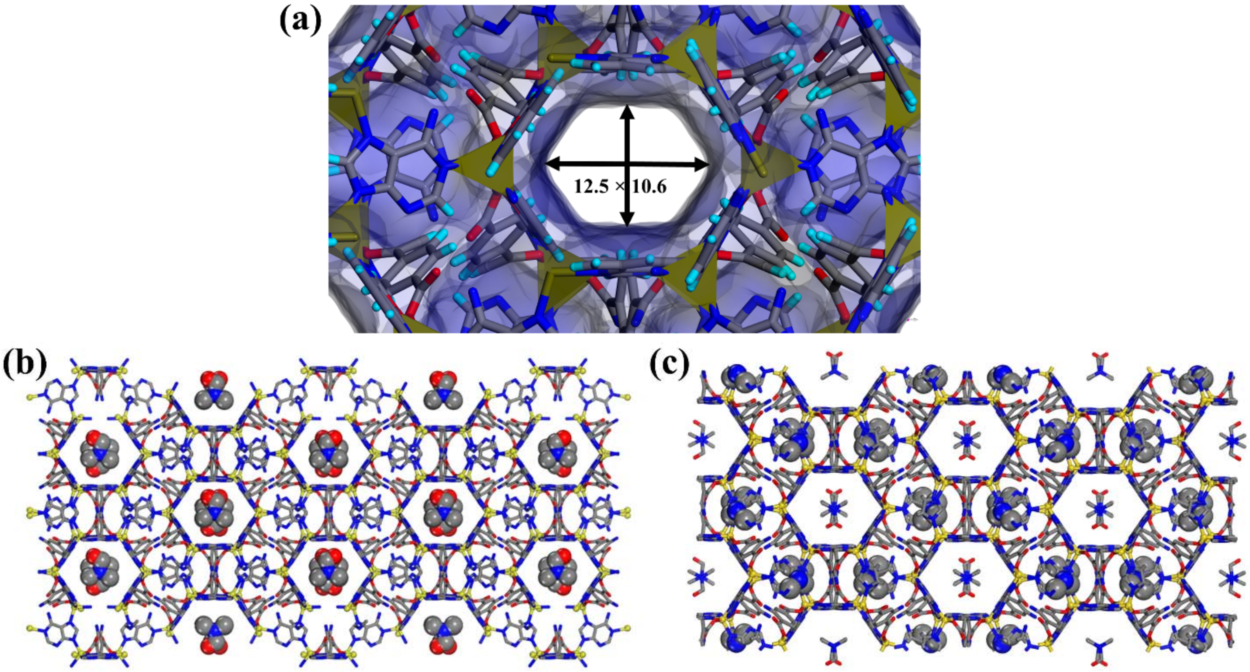

Figure S13. (a) Pore dimensions of **KBM-1** after considering VDW interactions. (b) Packing diagram showing DMF solvent molecules (CPK model) inside the pore. (c) Dimethyl ammonium cations sitting inside the interlayer space of the framework.

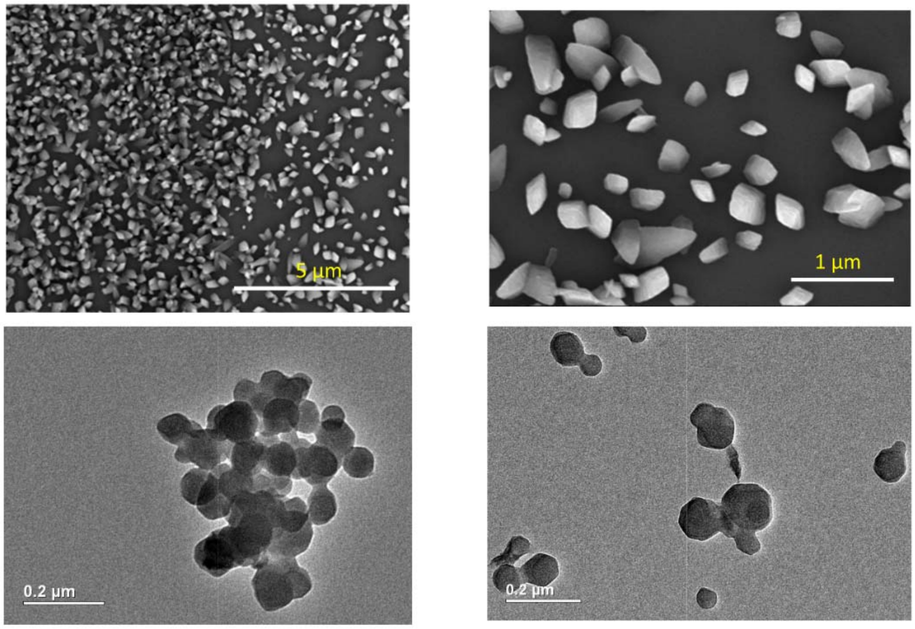

Figure S14. SEM (top) and TEM (bottom) images for **KBM-1** particles.

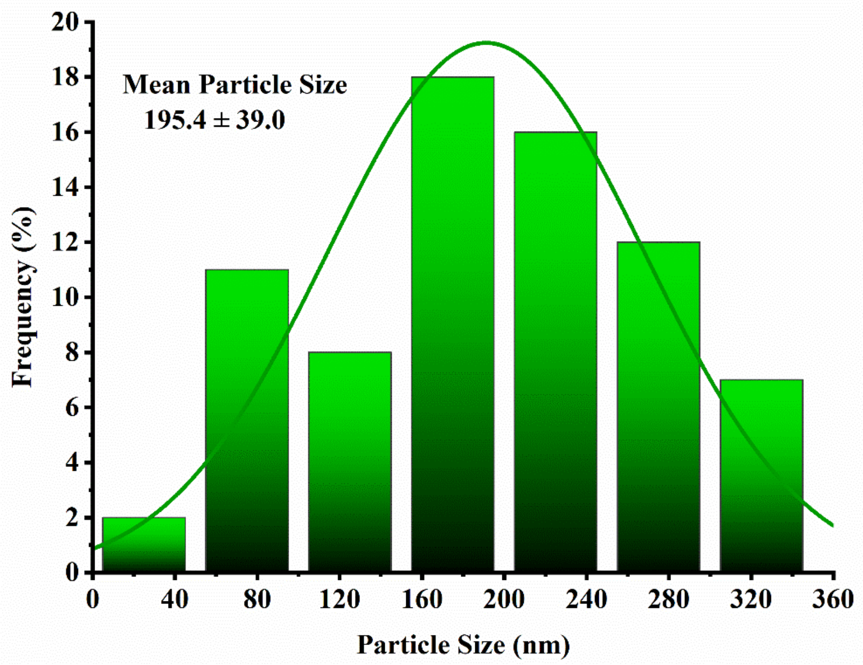

Figure S15. Particle size statistics analysis of **KBM-1**.

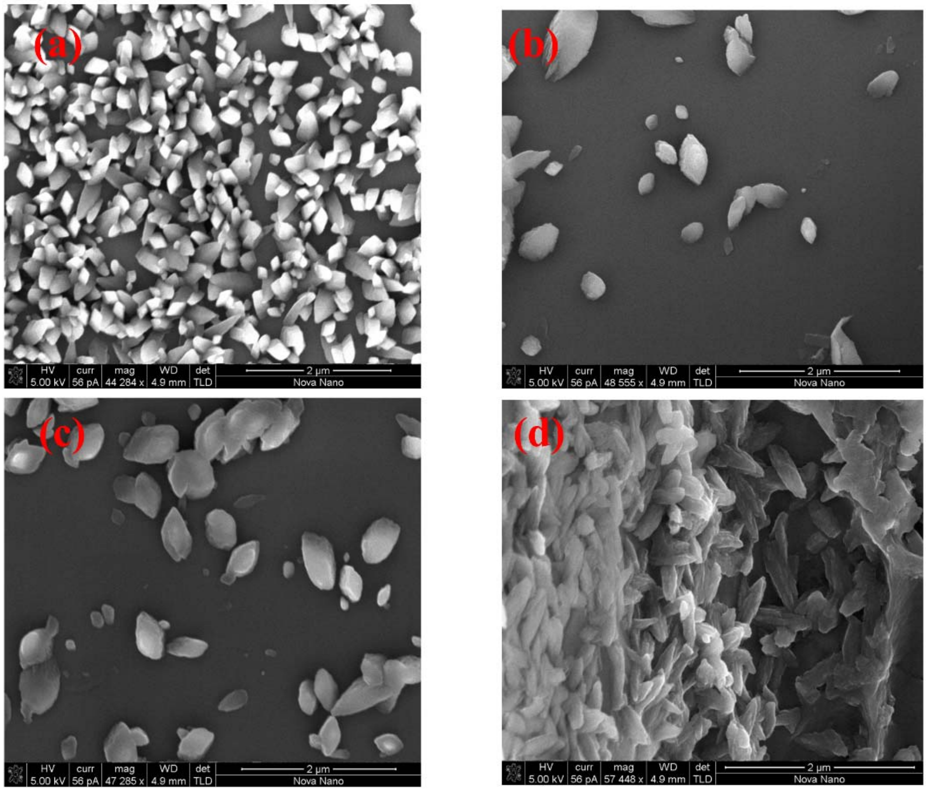

Figure S16. SEM images of **KBM-1** particles after incubation (16 hr) in (a) water, (b) media, (c) at pH 7 and (d) at pH 8.

Table S2. Crystal data and structure refinements for **KBM-2**.

Identification code	**KBM-2**
Empirical formula	'C_{82} H_{69} N_{26} O_{37} Zn_{8}'
Formula weight	2533.78
Temperature (K)	150(2)
Radiation	Mo-Kα
Wavelength (λ)	0.71073
Crystal system	Triclinic
Space group	*P*-1
a [Å]	17.716(9)
b [Å]	23.726(13)
c [Å]	25.856(15)
α [°]	115.39(2)

β [°]	92.62(4)
γ [°]	90.210(17)
Volume [Å^3]	9805(9)
Z	2
Density (calculated)[Mg m^{-3}]	0.8581
Absorption coefficient [mm^{-1}]	1.012
F(000)	2564
Refl. used [I >2σ(I)]	9640
Independent reflections	31589
R_{int}	0.1611
Refinement method	full-matrix least squares on F^2
GOF	0.9050
Final R indices [I>2σ(I)]	R_1 = 0.0864; wR_2 = 0.1916
R indices (all data)	R_1 = 0.2017; wR_2 = 0.3004

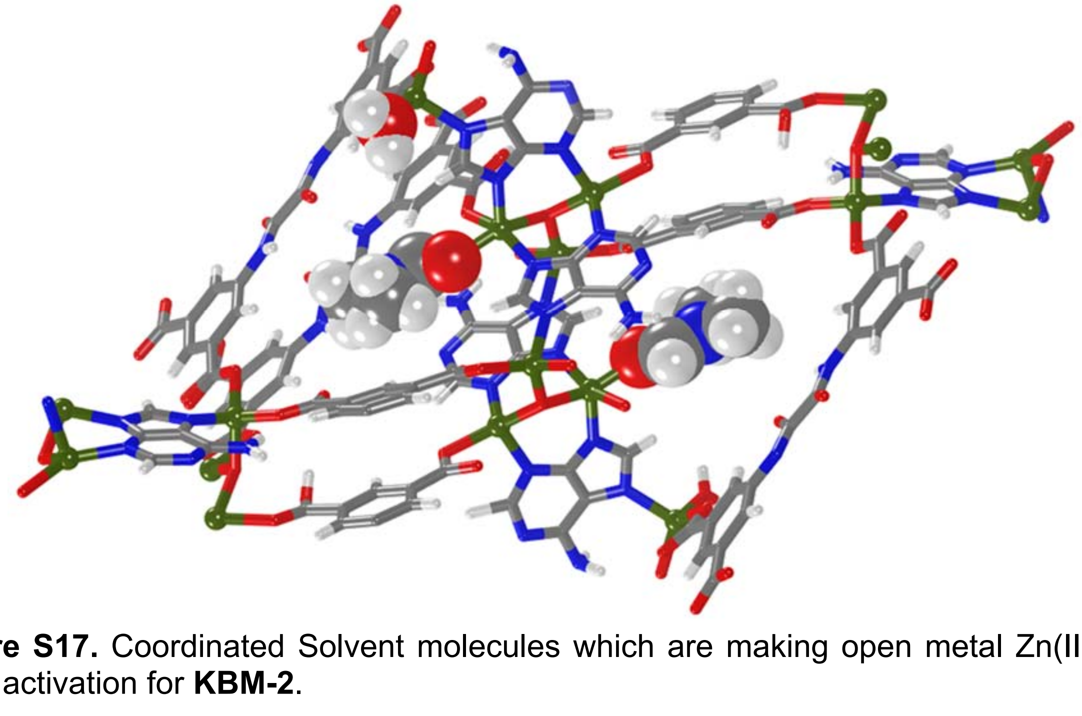

Figure S17. Coordinated Solvent molecules which are making open metal Zn(II) sites upon activation for **KBM-2**.

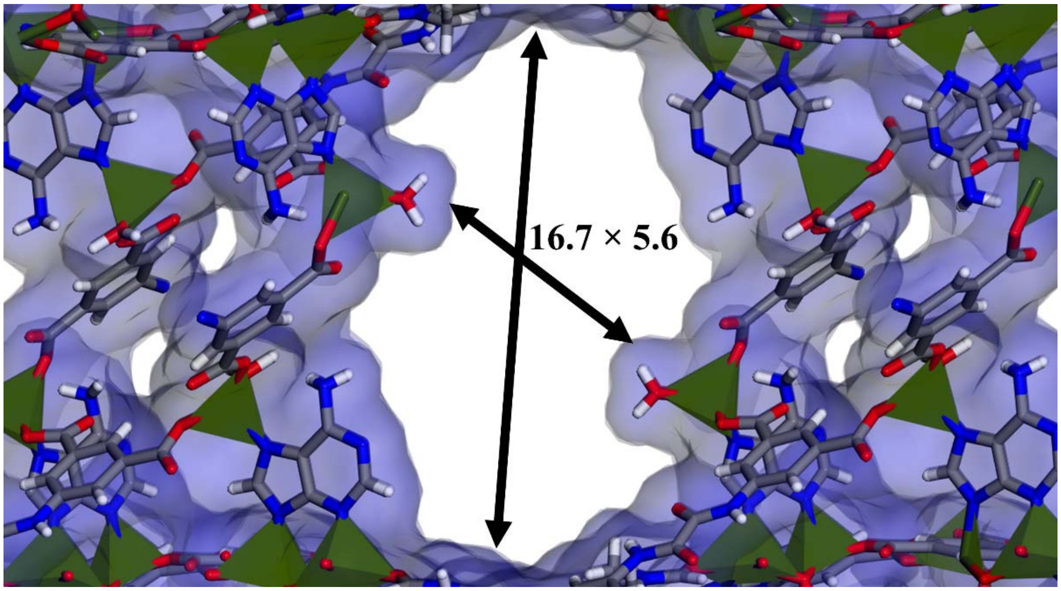

Figure S18. Pore dimensions of **KBM-2** after considering VDW interactions.

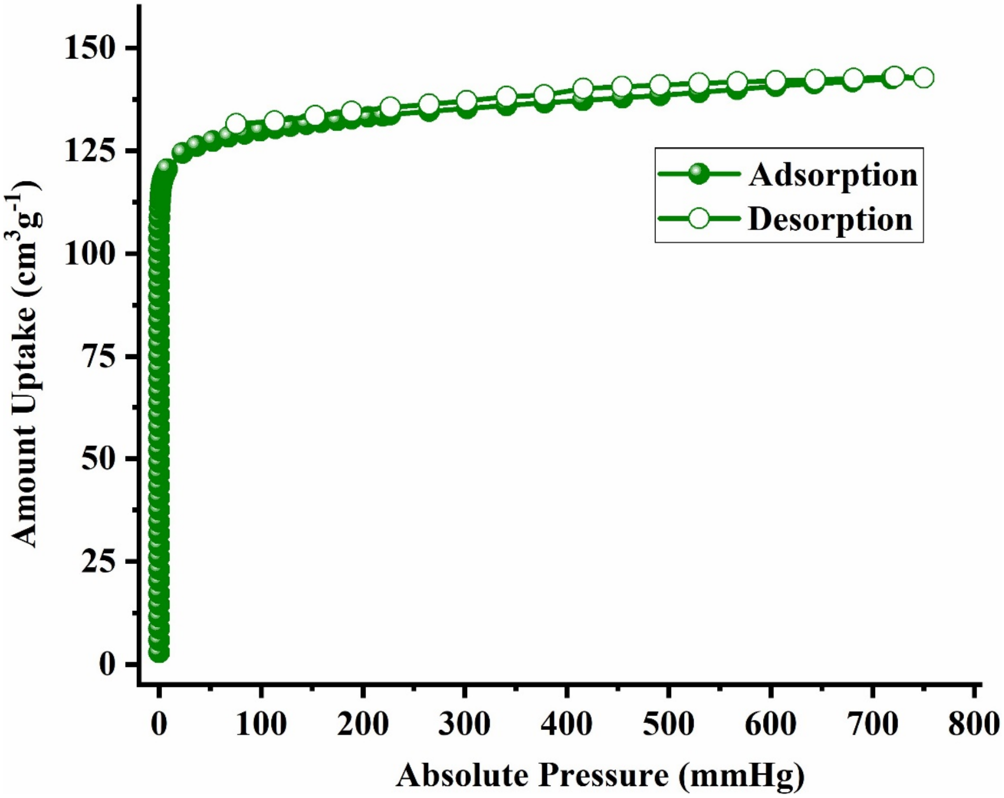

Figure S19. N_2 sorption at 77 K of **KBM-2**.

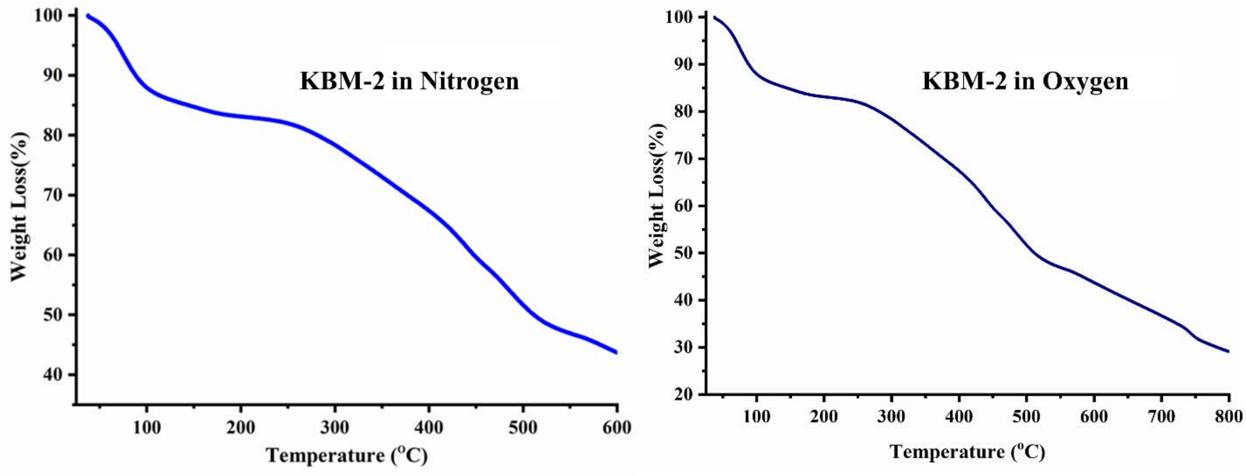

Figure S20. Thermo gravimetric analysis (TGA) data of the as synthesized **KBM-2**.

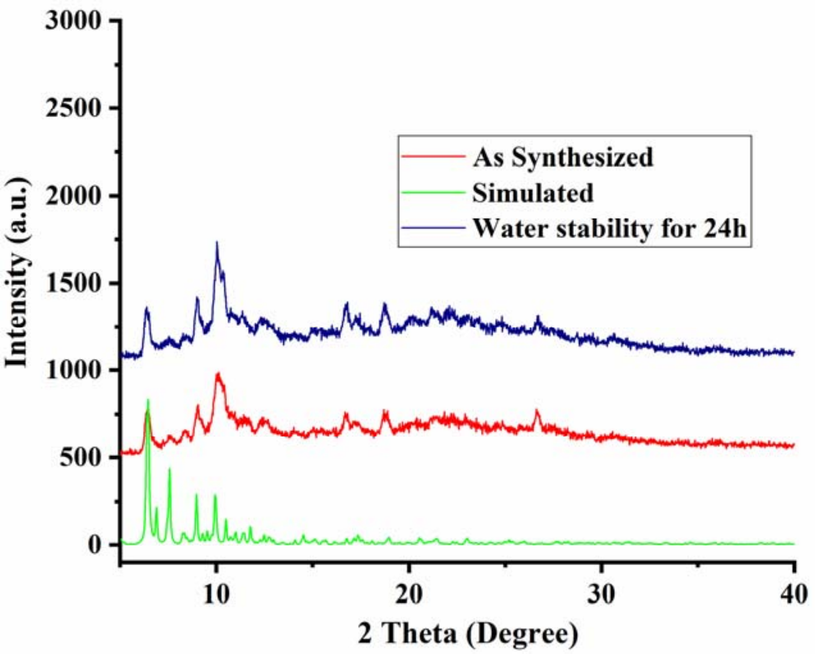

Figure S21. Comparison of the PXRD pattern of **KBM-2** as synthesized (black) with its water stability for 24 hours (red).

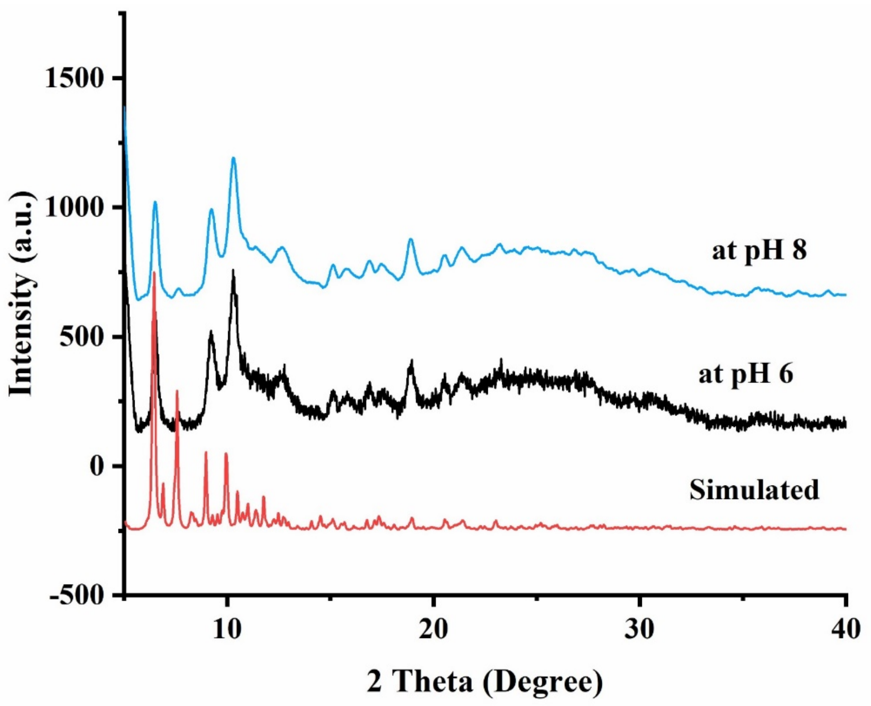

Figure S22. PXRD patterns of **KBM-2**: chemical stability test towards acidic and basic conditions for 24 hours. PBS concentration = 10mM, pH adjusted with HCl.

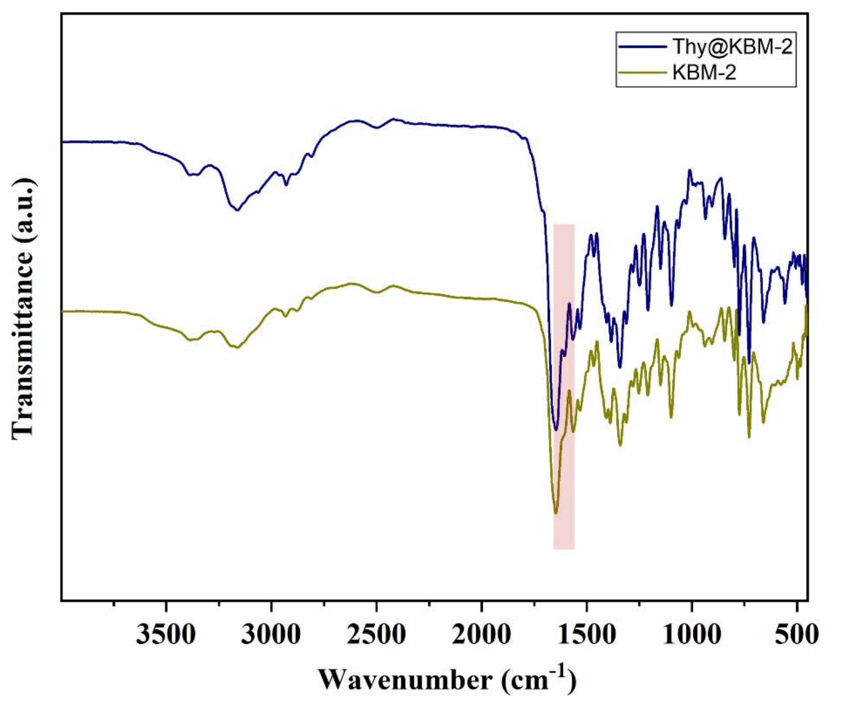

Figure S23. FTIR spectra of **KBM-2** and Thy@**KBM-2**.

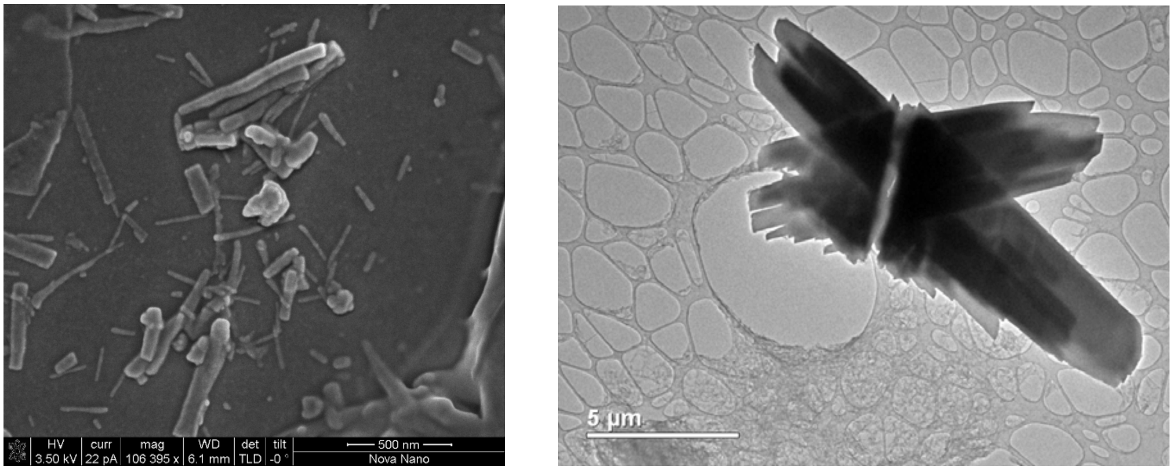

Figure S24. SEM (left) and TEM (right) images for **KBM-2**.

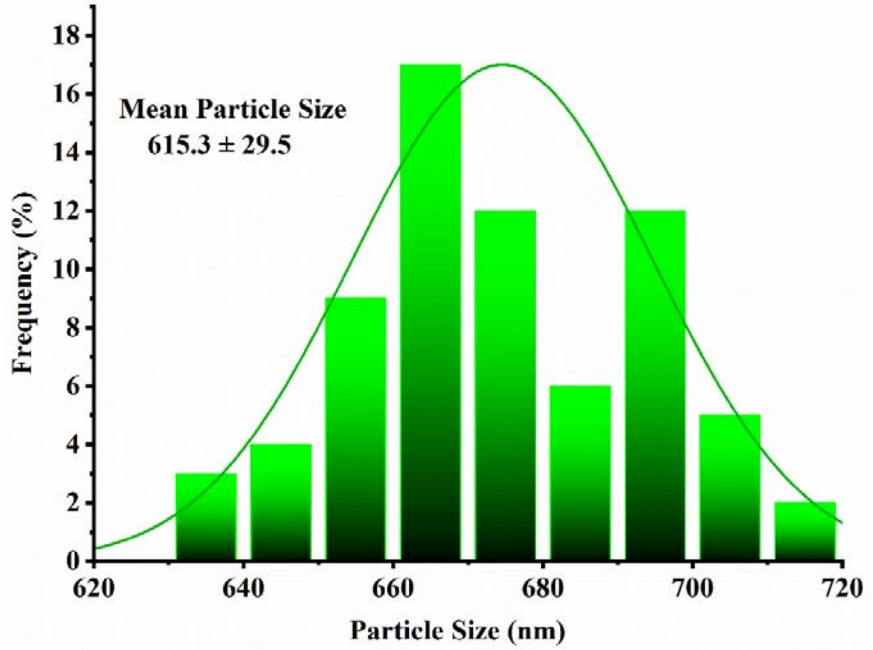

Figure S25. Particle size statistics analysis of **KBM-2**.

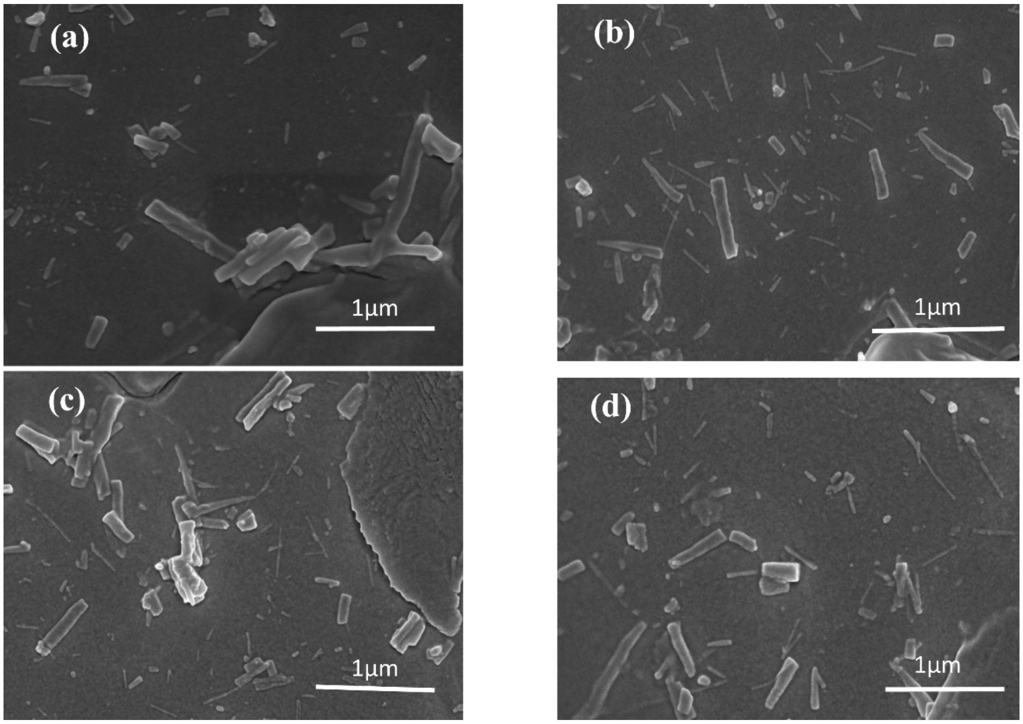

Figure S26. SEM images of **KBM-2** particles after incubation (16 hr) in (a) water, (b) media, in PBS (c) pH 7 and (d) in pH 8.

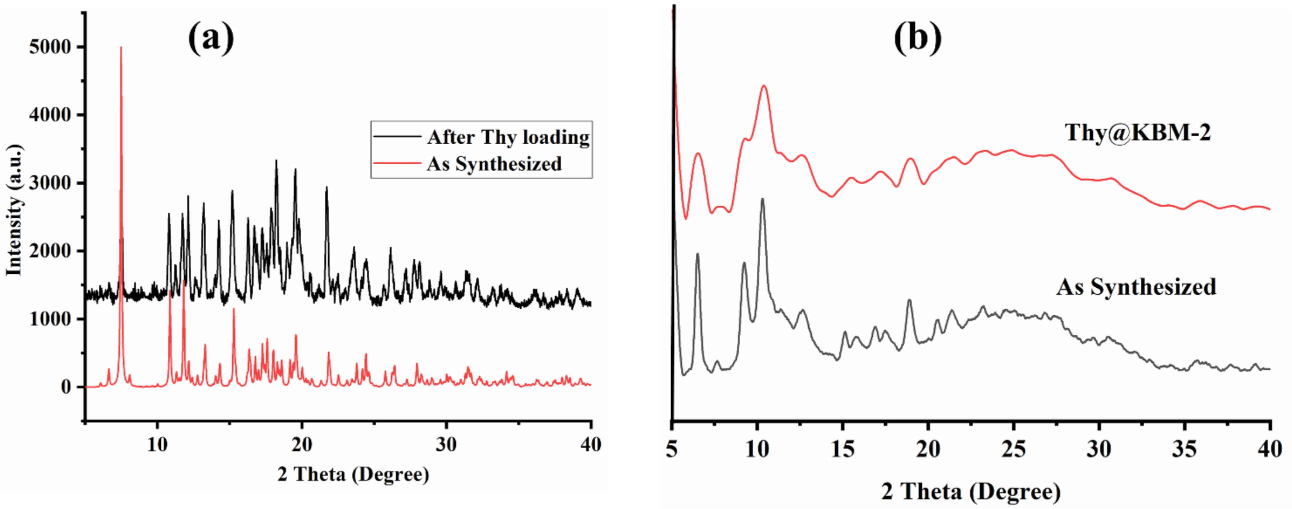

Figure S27. PXRD patterns of (a) **KBM-1** and (b) **KBM-2** after Thy loading experiments.

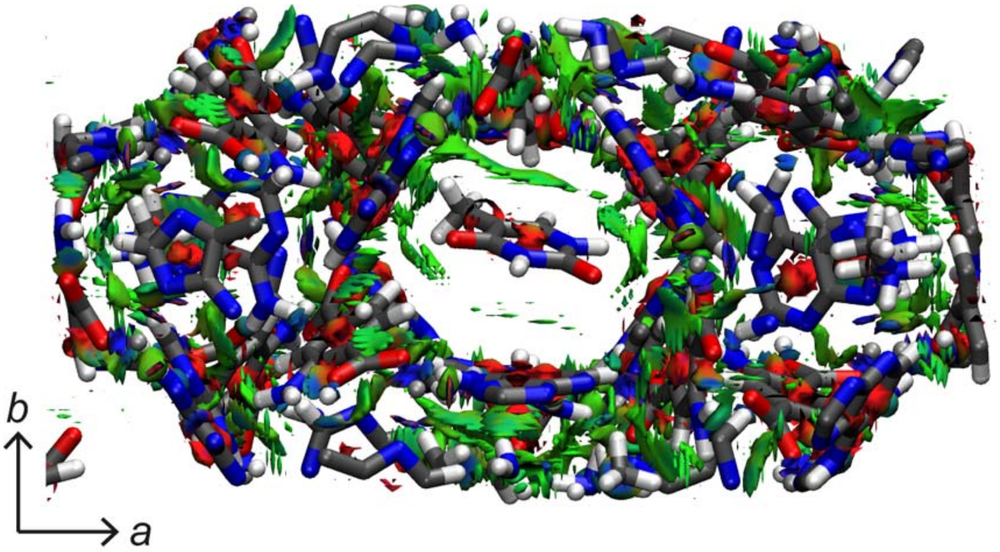

Figure S28. Plots of the NCI surfaces of Thy@**KBM-1**. Blue, green, and red regions indicate strong attractive interaction, weak interaction, repulsive interactions, respectively. The plotted range is -0.05 < sign(λ_2) < 0.05, and the plotted isosurface of reduced density gradient is 0.5. The gray, white, red, blue, and silver of atoms indicate C, H, O, N, and Zn, respectively.

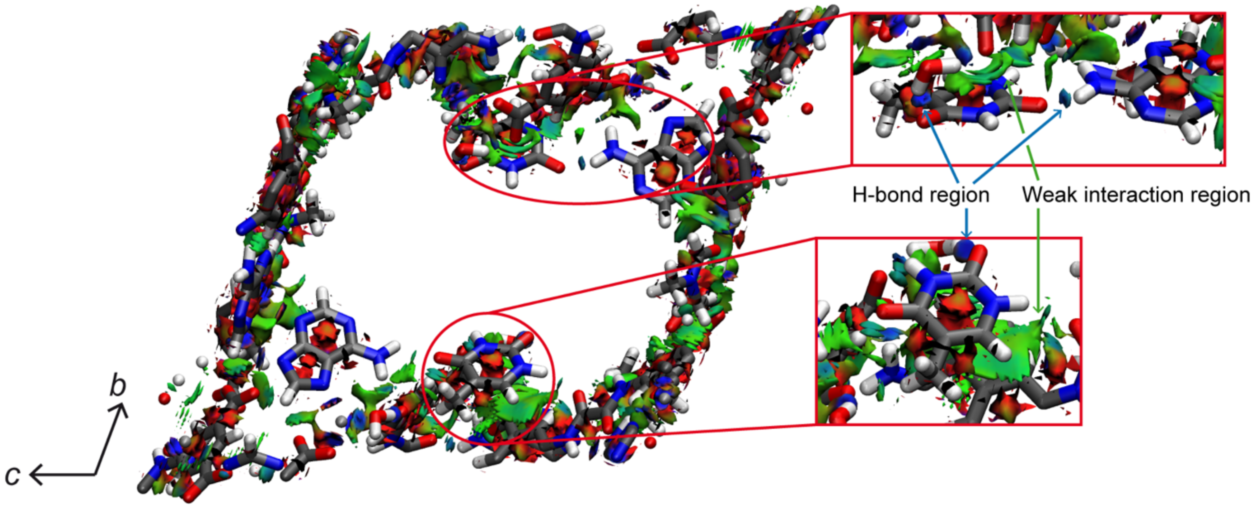

Figure S29. Plots of the NCI surfaces of Thy@**KBM-2**. Blue, green, and red regions indicate strong attractive interaction, weak interaction, repulsive interactions, respectively. The plotted range is -0.05 < sign (λ_2) < 0.05, and the plotted isosurface of reduced density gradient is 0.5. The gray, white, red, blue, and silver of atoms indicate C, H, O, N, and Zn, respectively.

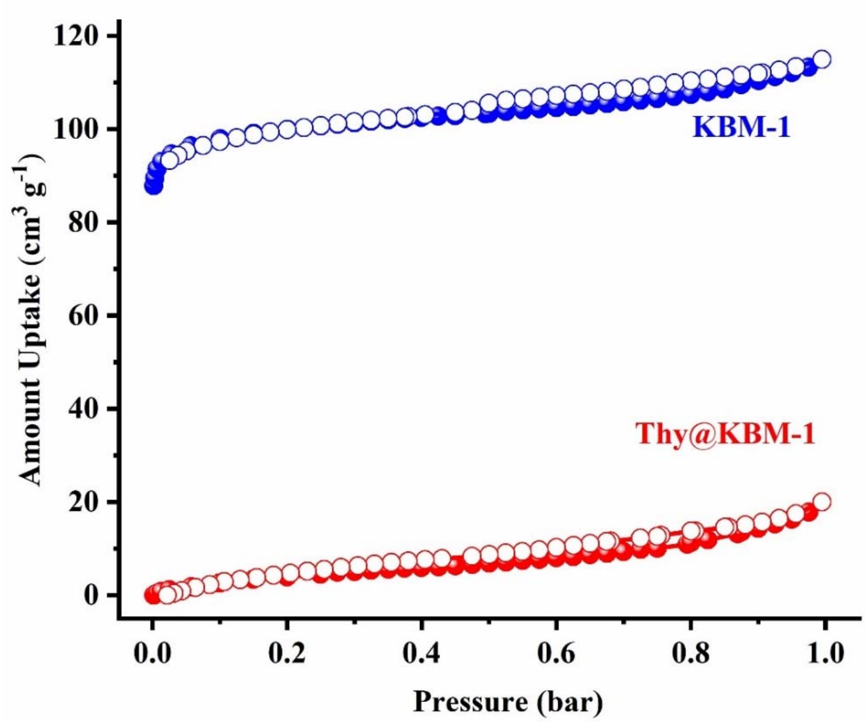

Figure S30. Comparison of N_2 sorption isotherm of **KBM-1** and Thy loaded **KBM-1**.

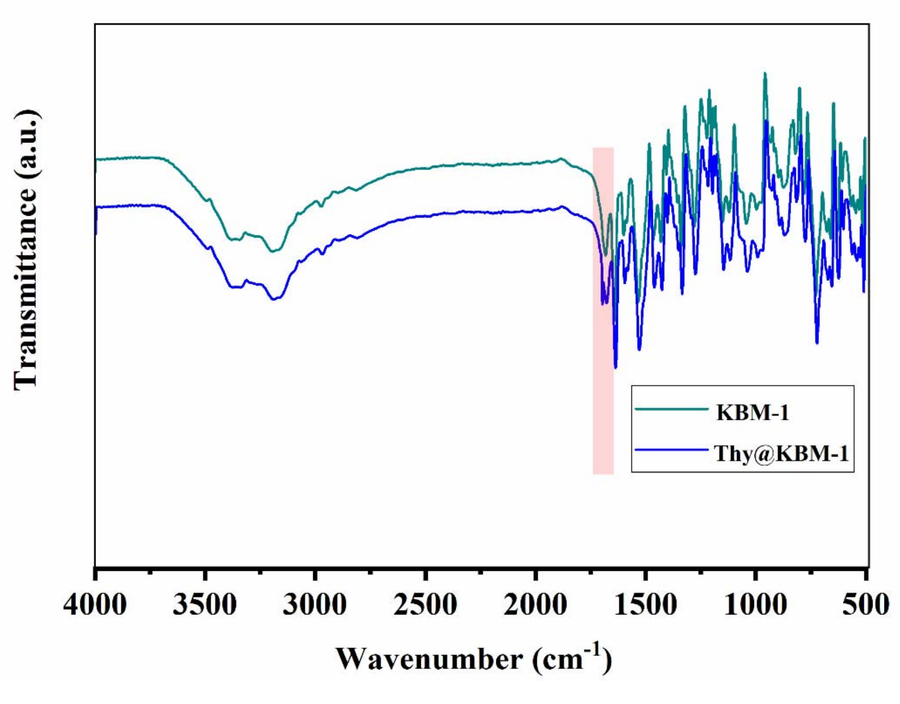

Figure S31. Comparison of the FT-IR spectra of **KBM-1** and Thy@ **KBM-1**.

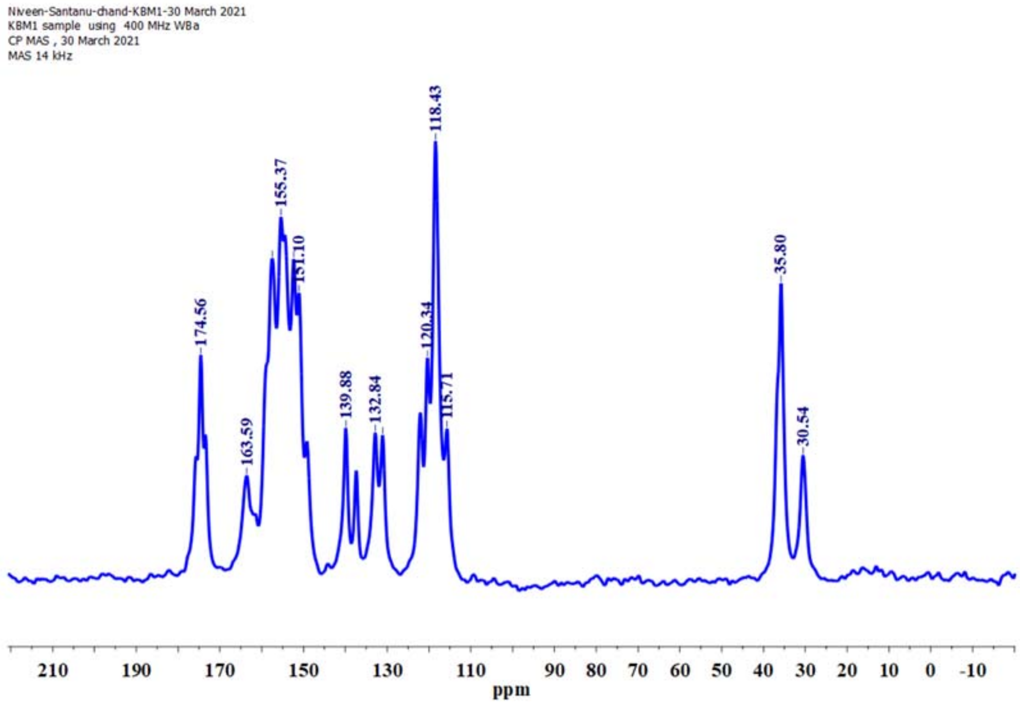

Figure S32. Solid state ^{13}C NMR spectra (CP-MAS) of **KBM-1.**

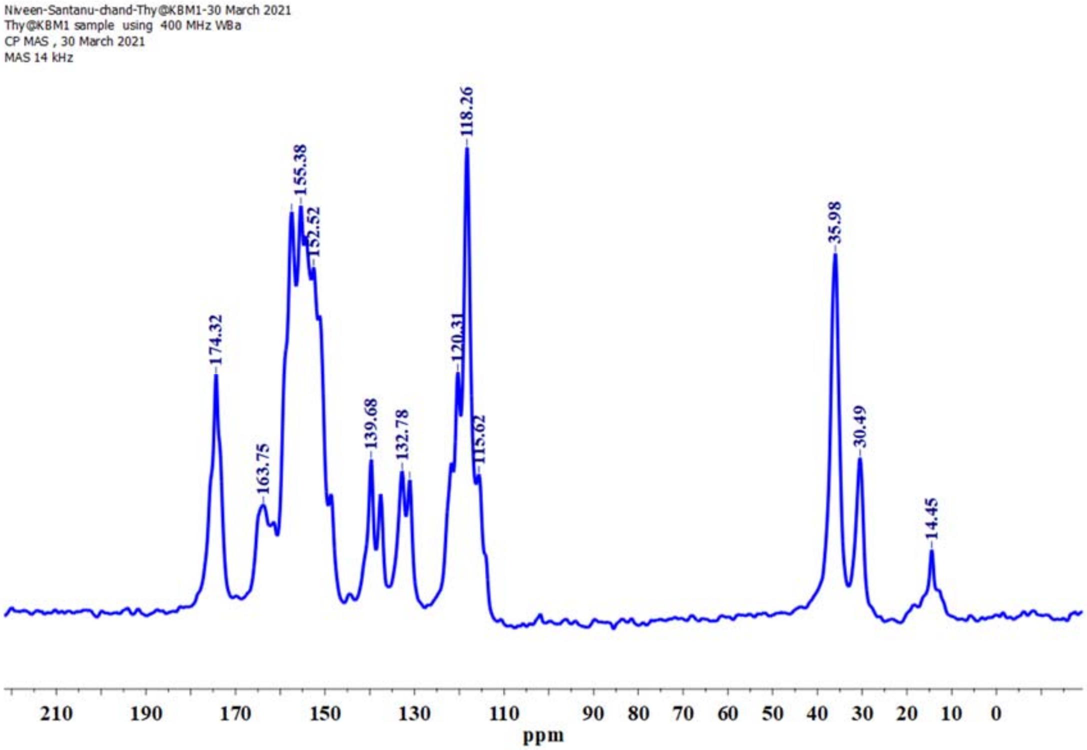

Figure S33. Solid state ^{13}C NMR spectra (CP-MAS) of Thy@**KBM-1**.

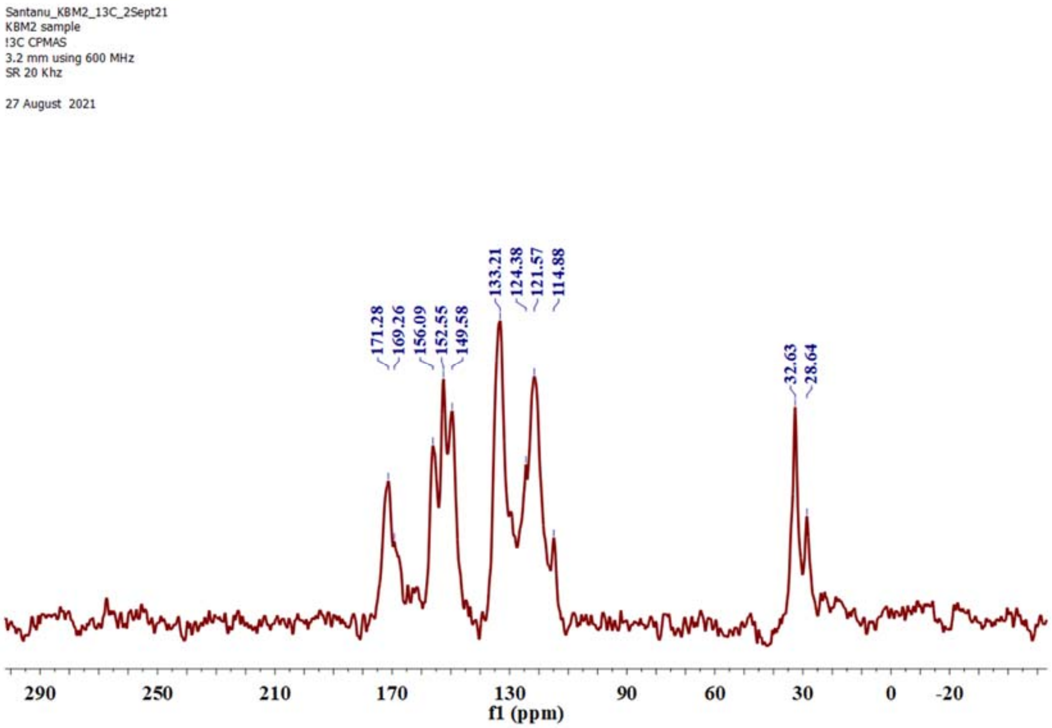

Figure S34. Solid state ^{13}C NMR spectra (CP-MAS) of **KBM-2.**

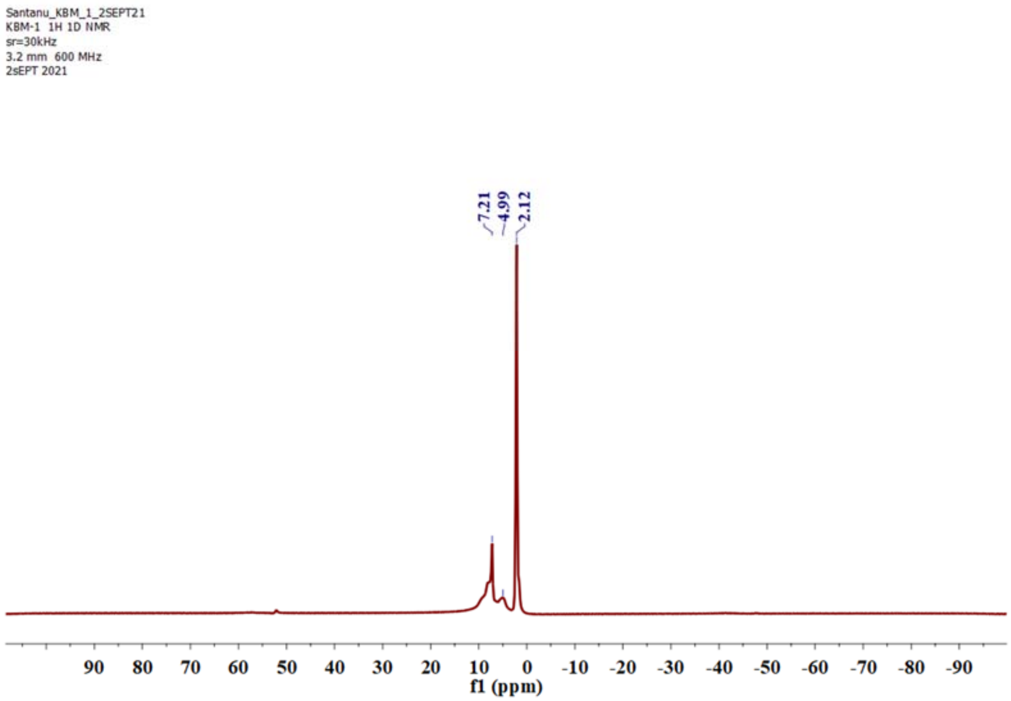

Figure S35. Solid state ^{1}H NMR spectra (CP-MAS) of **KBM-1.**

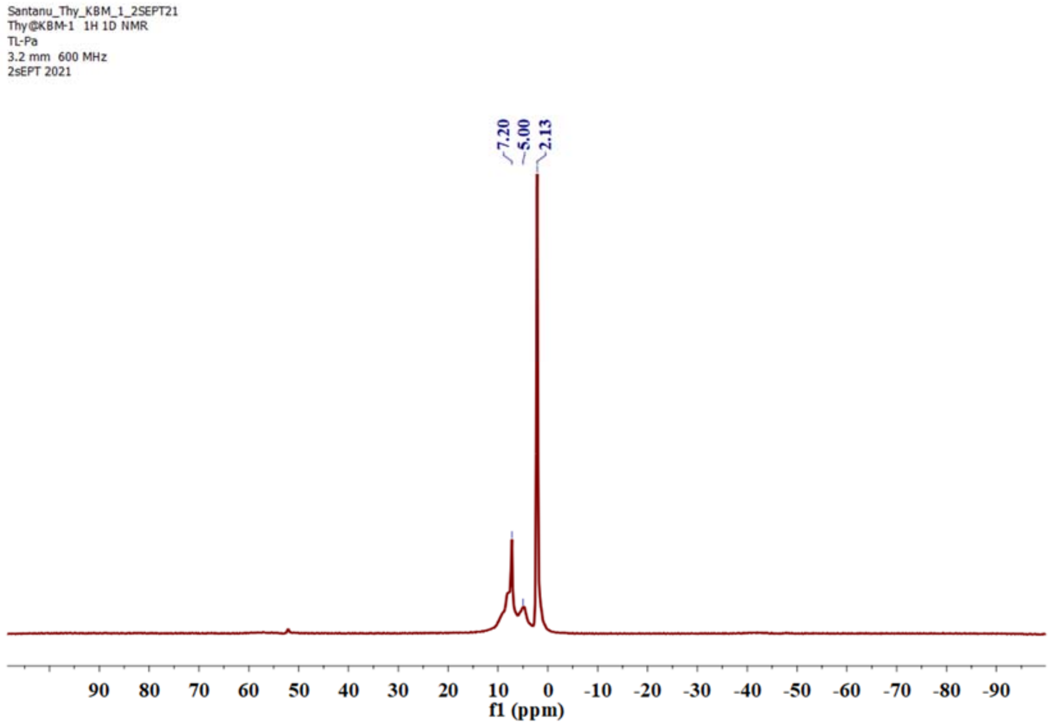

Figure S36. Solid state ^{1}H NMR spectra (CP-MAS) of Thy@**KBM-1.**

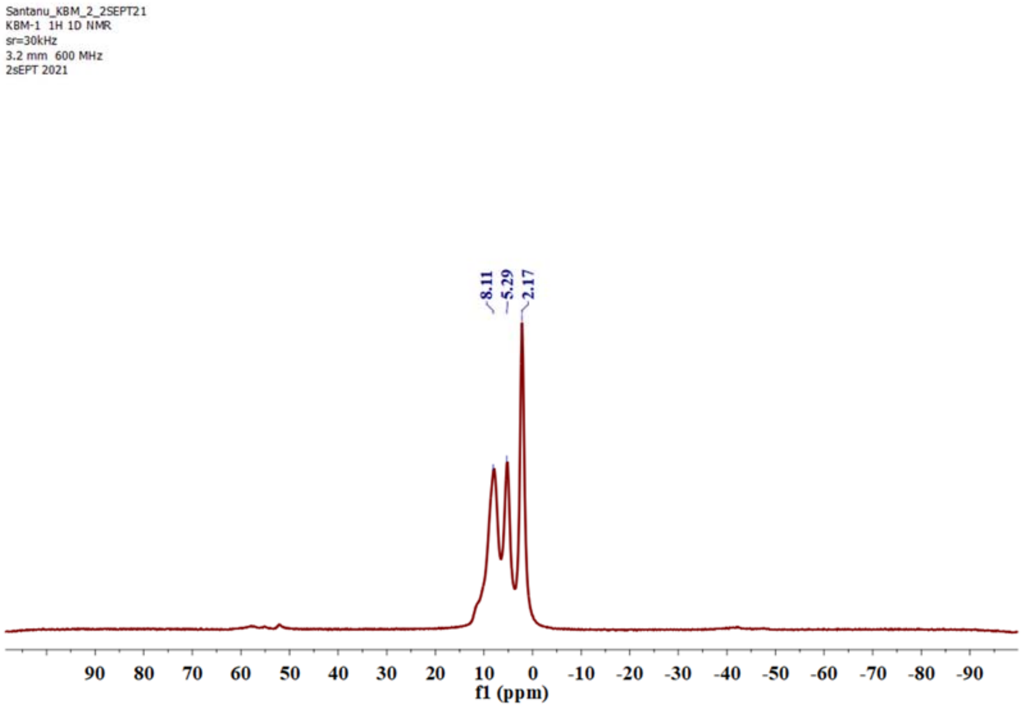

Figure S37. Solid state ^{1}H NMR spectra (CP-MAS) of **KBM-2.**

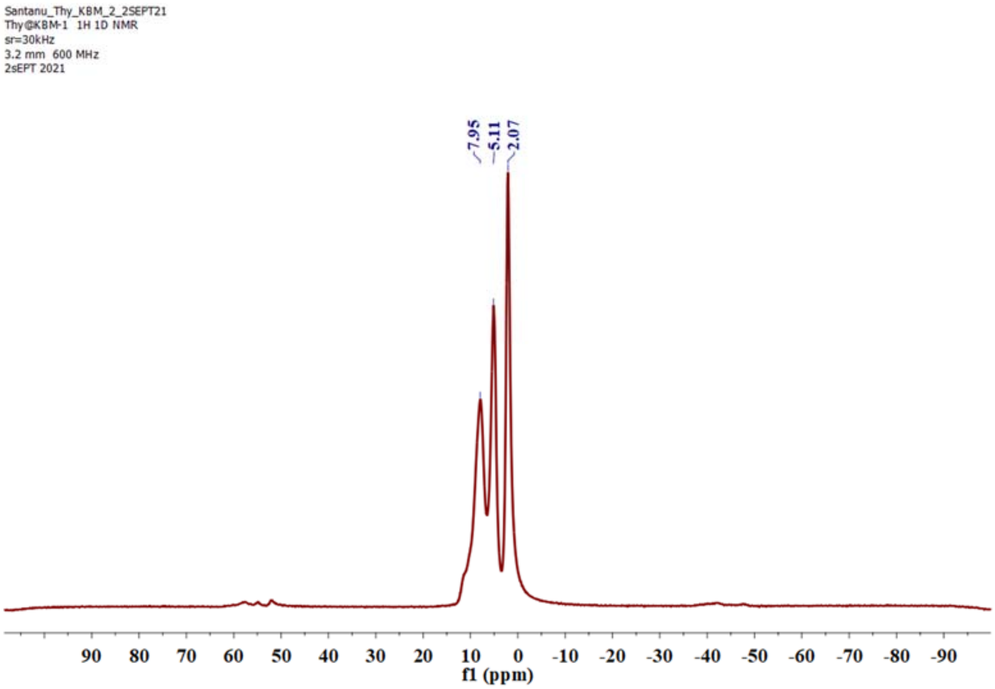

Figure S38. Solid state ^{1}H NMR spectra (CP-MAS) of Thy@**KBM-2**.

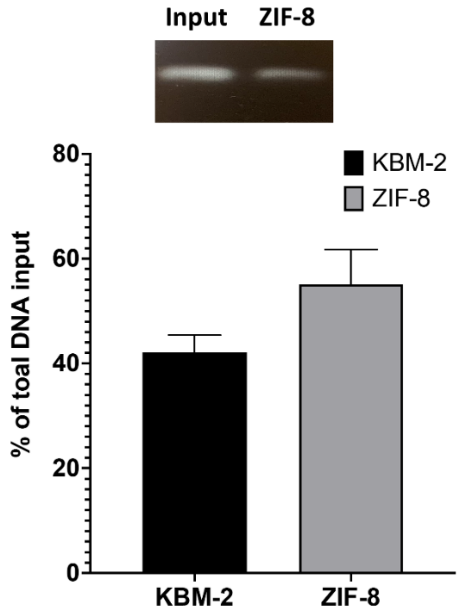

Figure S39. Analysis of **KBM-2** and ZIF-8 loading efficiency of Anti-PCNA aptamer.

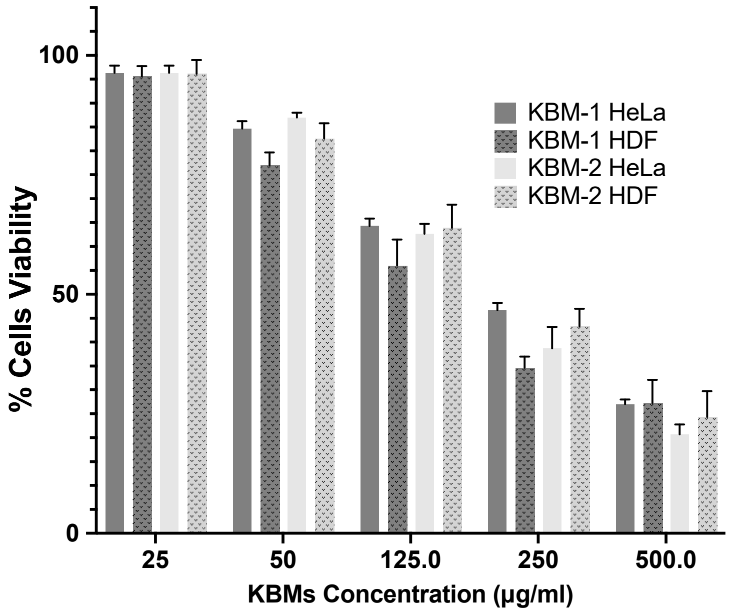

Figure S40. Cytotoxicity analysis of **KBM-1** and **KBM-2** to HeLa and HDF cells using MTT assay (n = 3).

Table S3. Zeta potential analysis of **KBM-1**, **KBM-2** and ssDNA **KBM-2**.

KBM	Zeta potential
**KBM-1**	- 6.8
**KBM-2**	0.47
**ssDNA@KBM-2**	-11.34

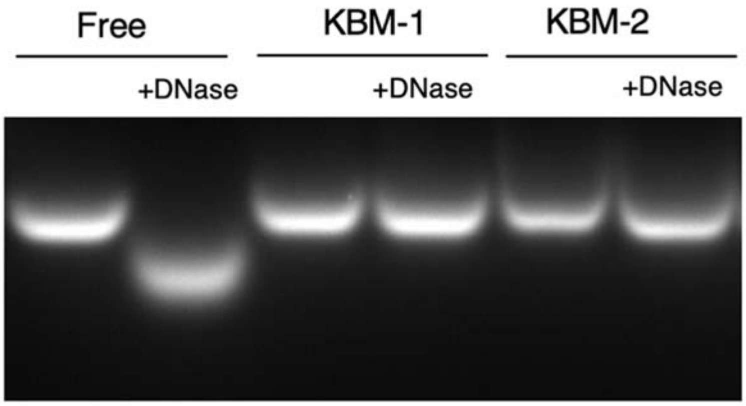

Figure S41. Gel agarose analysis of DNase I digestion reaction of ssDNA loaded to **KBM-1** and **KBM-2**.

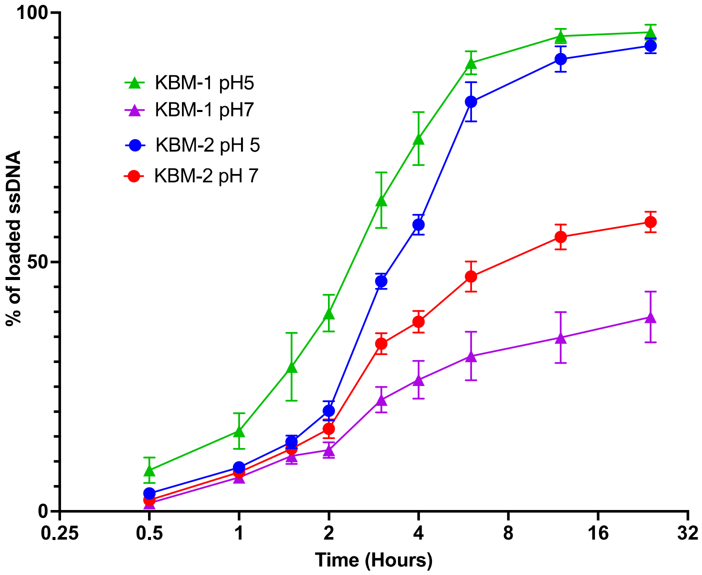

Figure S42. ssDNA release from **KBM-1** and **KBM-2** over 24 hours incubation in Tris-HCl buffer at pH 7 and pH 5.5.

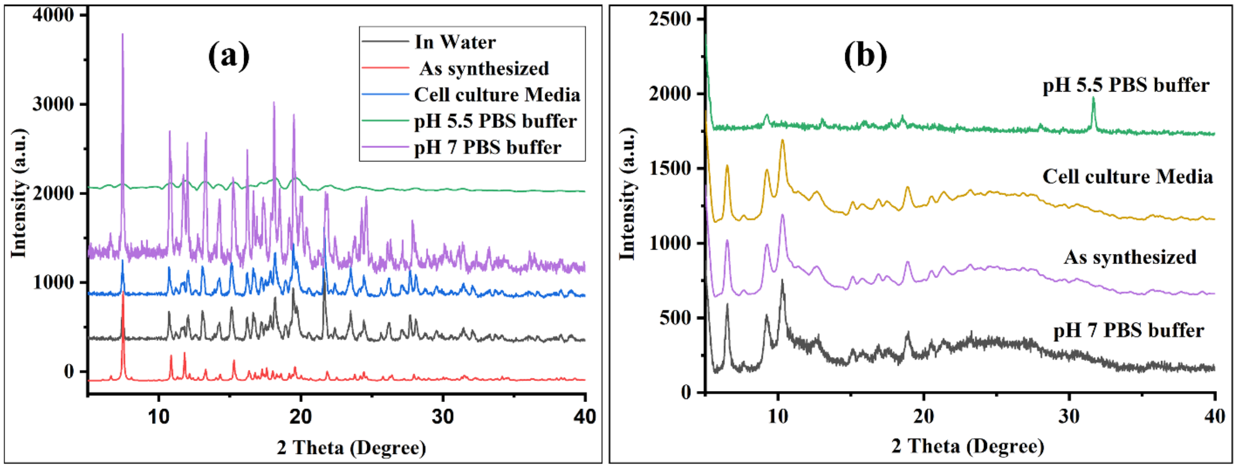

Figure S43. PXRD patterns of **KBM-1** (a) and **KBM-2** (b) chemical stability test towards different acidic media conditions for 24 hours. PBS concentration = 10mM, pH adjusted with HCl.

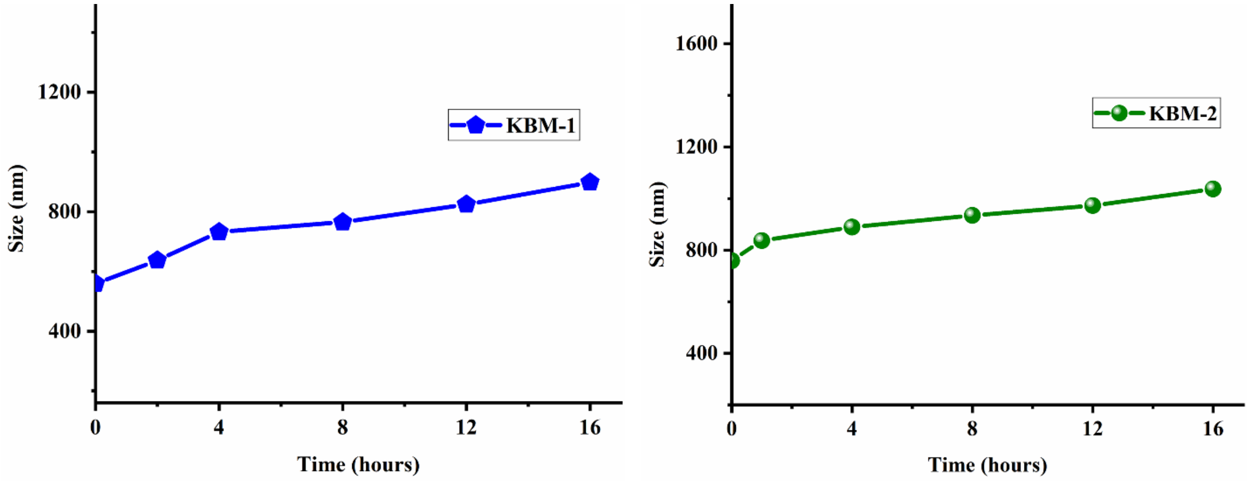

Figure S44. Dynamic light scattering (DLS) study of **KBM-1** and **KBM-2** in aqueous media (16 hr).

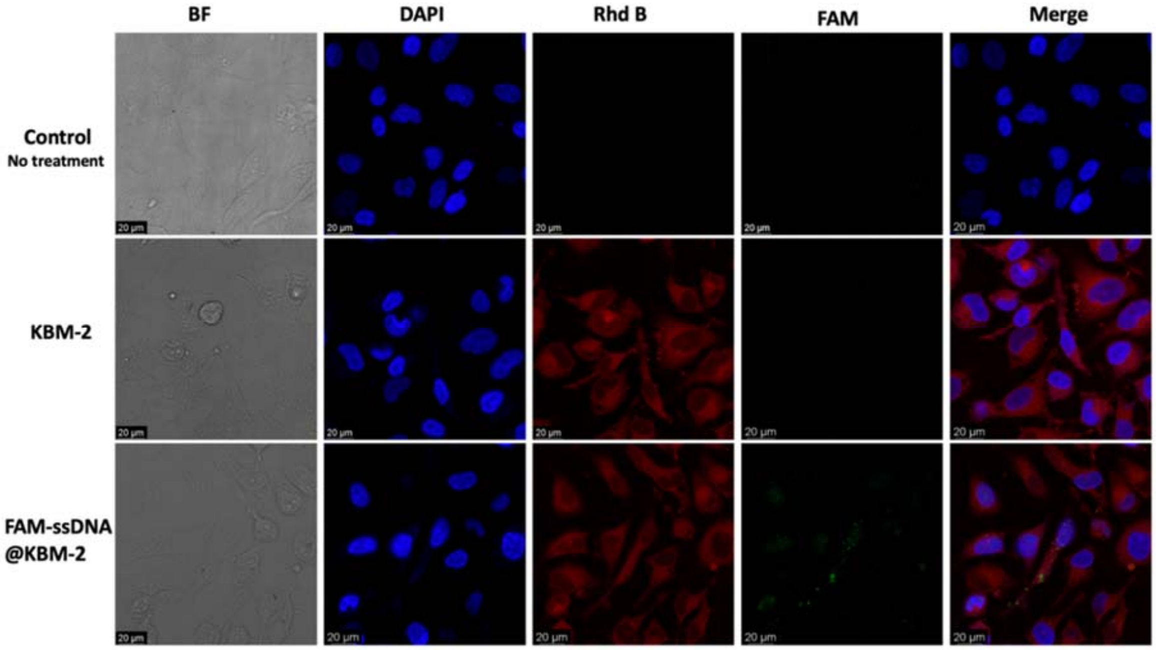

Figure S45. Analysis of **KBM-2** cellular uptake and ssDNA delivery using confocal microscopy.

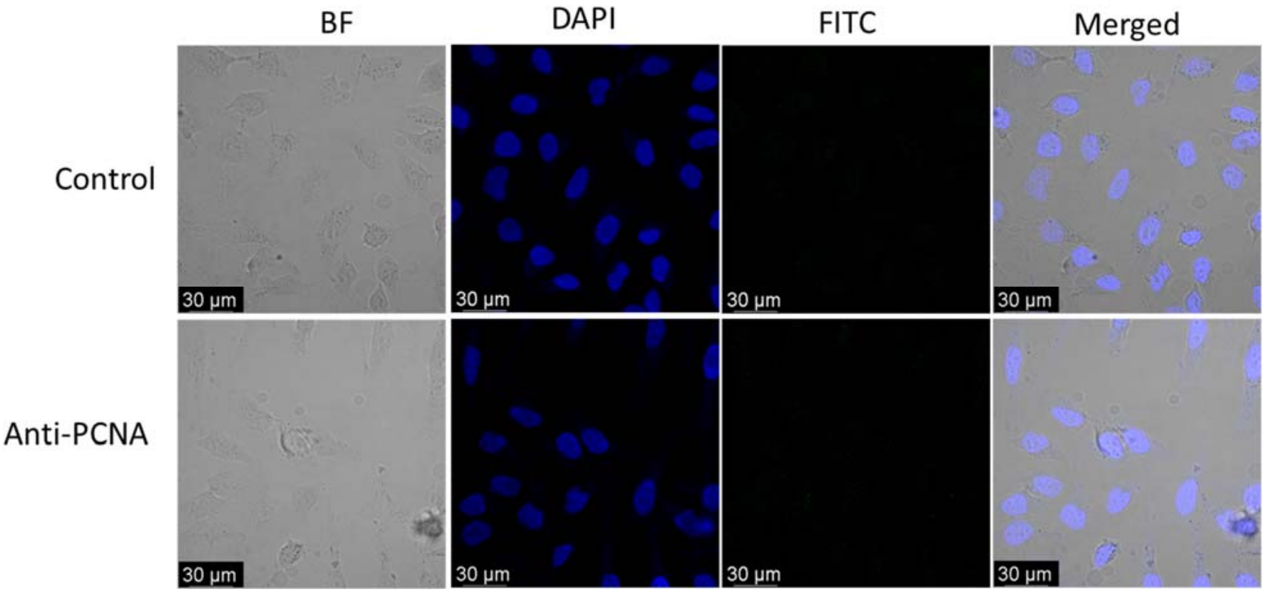

Figure S46. Analysis of FAM labelled Anti-PCNA cellular uptake using confocal microscopy.

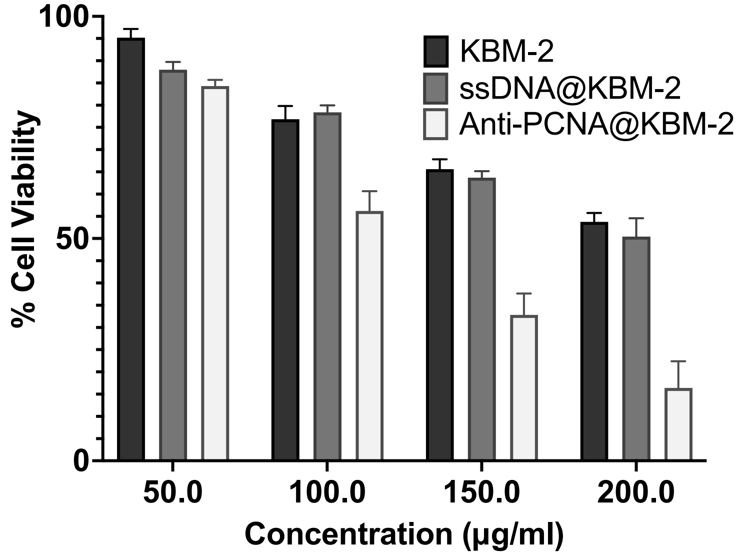

Figure S47. Cytotoxicity evaluation of **KBM-2**, cDNA@**KBM-2** and Anti-PCNA@**KBM-2** using MTT assay (*n* = 3).

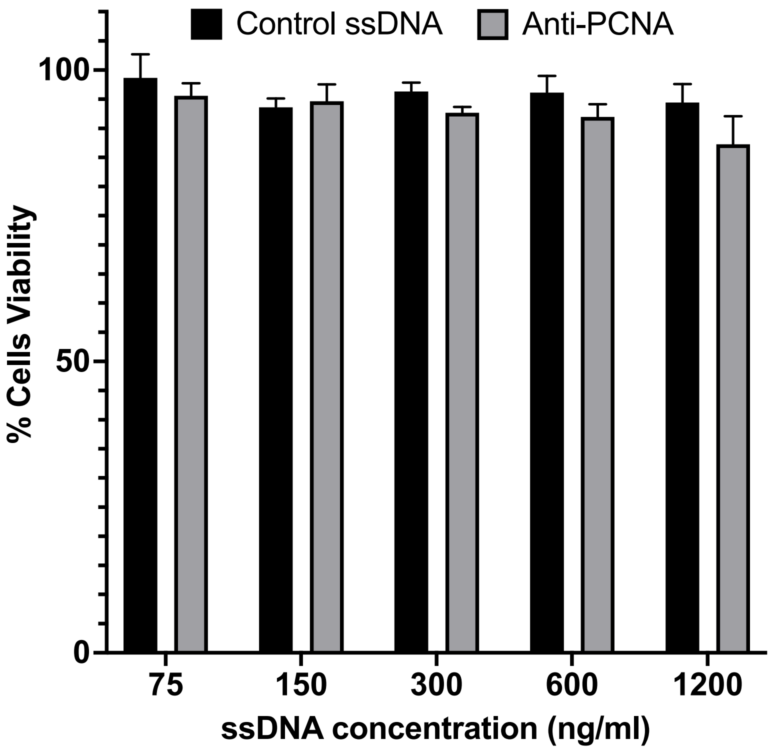

Figure S48. Cytotoxicity analysis of control ssDNA and Anti-PCNA to HeLa MTT assay (*n* =3).

Table S4. Selected bond distances and angle observed in **KBM-1**

Zn1 — O1	1.943(2)	Zn2 — N2	2.009(2)
Zn1 — N1	1.977(2)	Zn2 — N9	2.023(2)
Zn1 — N6	1.997(2)	Zn3 — O5	1.947(2)
Zn1— N12	2.009(2)	Zn3 — N11	1.977(2)
Zn2 — O4	1.9350(19)	Zn3 — N7	1.991(2)
Zn2 — N16	1.974(2)	Zn3 — N17	2.001(2)

O1— Zn1— N1	108.17(9)	N16 — Zn2 — N9	112.78(9)
O1 — Zn1— N6	114.56(10)	N2 — Zn2 — N9	107.27(9)
N1 — Zn1— N6	113.25(9)	O4 — Zn2 — N9	114.27(10)
O1— Zn1— N12	101.37(9)	O5— Zn3 — N11	107.69(10)
N1 — Zn1 — N12	111.03(9)	O5 — Zn3 — N7	107.96(10)
N6 — Zn1 — N12	107.84(9)	N11 — Zn3 — N7	109.92(10)
O4 — Zn2 — N16	109.27(9)	O5 — Zn3 — N17	103.48(9)
O4 — Zn2 — N2	103.54(8)	N11 — Zn3 — N17	113.23(9)
N16 — Zn2 — N2	109.18(9)	N7— Zn3 — N17	114.05(10)

References:

1) Lü, J.; Perez-Krap, C.; Suyetin, M.; Alsmail, N. H.; Yan, Y.; Yang, S.; Lewis, W.; Bichoutskaia, E.; Tang, C. C.; Blake, A. J.; Cao, R.; Schröder, M. *J. Am. Chem. Soc.* **2014**, *136*, 12828.
2) SAINT. Bruker AXS. Inc, Madison, Wisconsin, USA, **2014**.
3) SADABS. G. M. Sheldrick. University of Gottingen, Germany, **2008**.
4) XPREP, 5.1 ed. Siemens Industrial Automation Inc., Madison, WI, **1995**.
5) Sheldrick, G. M. *SHELXTL ™Reference Manual*: version 5.1, Bruker AXS, Madison, WI, **1997**.
6) Sheldrick, G. M. Crystal Structure Refinement with SHELXL. *Acta Cryst C*, **2015**, *71*, 3–8.
7) WinGX. L. J. Farrugia, *J. Appl. Cryst*. **2012**, *45*, 849-854.
8) Spek, A. L. Single-crystal Structure Validation with the Program PLATON. *J. Appl. Crystallogr*. **2003**, *36*, 7−13.
9) M. Elstner, D. Porezag, G. Jungnickel, J. Elsner, M. Haugk, Th. Frauenheim, S. Suhai, and G. Seifert, *Phys. Rev. B*, **1998**, *58*, 7260–7268.

10) Y. Yang, H. Yu, D. York, Q. Cui, M. Elstner, *J. Phys. Chem. A* **2007**, *111*, 10861–10873.
11) B. Aradi, B. Hourahine, and Th. Frauenheim, *J. Phys. Chem. A*, **2007**, *111*, 5678–5684.
12) M. Gaus, A. Goez, M. Elstner, *J. Chem. Theory Comput.*, **2013**, *9*, 338–354.
13) S. Grimmea, J. Antony, S. Ehrlich, H. Krieg, J. *Chem. Phys.*, **2010**, *132*, 154104.
14) E. R. Johnson, S. Keinan, P. M-Sanchez, J. Contreras-García, A. J. Cohen, W. Yang, *J. Am. Chem. Soc*. **2010**, *132*, 6498–6506.
15) J. Contreras-García, E. R. Johnson, S. Keinan, R. Chaudret, J.-P. Piquemal, D. N. Beratan, W. Yang, *J. Chem. Theo. Comput.* **2011**, *7*, 625–632.
16) W. Humphrey, A. Dalke, K. Schulten, *J. Mol. Graphics*. **1996**, *14*, 33–38.
17) An, J.; Geib, S. J.; Rosi, N. L. *J. Am. Chem. Soc*. **2009**, *131*, 8376.
18) Banerjee, R.; Phan, A.; Wang, B.; Knobler, C.; Furukawa, H.; O'Keeffe, M.; Yaghi, O. M. Science 2008, 319, 939−943.
19) E. Kowalska, F. Bartnicki, R. Fujisawa, P. Bonarek, P. Hermanowicz, T. Tsurimoto, K. Muszynska, W. Strzalka, *Nucleic Acids Res*. **2018***, 46*, 25–41.
